# Structural Analysis and Mechanical Performance of Industrial Conveyor Flight Bars Manufactured with Epoxy Matrix Composites Reinforced by Glass, Carbon, and Kevlar Fibers

**DOI:** 10.3390/polym18040433

**Published:** 2026-02-09

**Authors:** Antonio Henrique da Silva Bitencourt Junior, Maurício Maia Ribeiro, Douglas Santos Silva, Raí Felipe Pereira Junio, Sergio Neves Monteiro, Jean da Silva Rodrigues

**Affiliations:** 1 Materials Engineering Program, Federal Institute of Education, Science and Technology of Pará—IFPA, Avenida Almirante Barroso, 1155, Marco, Belém CEP 66093-020, PA, Brazil; antonio.bitencourt@ifpa.edu.br (A.H.d.S.B.J.); jean.rodrigues@ifpa.edu.br (J.d.S.R.); 2 Federal Institute of Education, Science and Technology of Pará—IFPA, Estrada do Icuí Guajará, Ananindeua CEP 67125-000, PA, Brazil; mauricio.maia@ifpa.edu.br; 3 Department of Materials Science, Military Institute of Engineering—IME, Praça General Tibúrcio, 80, Praia Vermelha, Urca, Rio de Janeiro CEP 22290-270, RJ, Brazil; raivsjfelipe@ime.eb.br (R.F.P.J.); sergio.neves@ime.eb.br (S.N.M.)

**Keywords:** polymer matrix composites, classical laminate theory, fiber reinforcement, industrial conveyor systems

## Abstract

Industrial conveyor systems commonly use steel flight bars, which can account for nearly 50% of the total system mass and significantly affect energy consumption. This study investigates epoxy matrix composites reinforced with glass, carbon, and Kevlar fibers as lightweight alternatives to steel flight bars. A multiscale analytical approach combining micromechanics, Classical Laminate Theory (CLT), and ply-level failure criteria is applied to evaluate the structural response under an industrial bending moment of 342.02 N·m. Tensile tests on vacuum-infused woven glass/epoxy laminates are used to validate micromechanical assumptions and calibrate elastic properties. Ply-wise analysis shows that carbon/epoxy laminates exhibit the lowest longitudinal stresses (≈43 MPa), followed by Kevlar/epoxy (≈53 MPa) and glass/epoxy (≈95 MPa), all well below their respective strength limits. Replacing steel flight bars (4.64 t) with composite alternatives reduces the moving mass to 0.68–0.82 t, corresponding to an 82–85% reduction. This mass reduction significantly lowers the required mechanical power, resulting in an estimated annual energy saving of R$ 8812.80 under continuous operation. Overall, the results demonstrate that polymer-matrix composite flight bars are structurally safe and energetically advantageous, with carbon/epoxy providing the highest mechanical efficiency.

## 1. Introduction

The demand for lightweight structural components has increased significantly across industrial sectors in which mass reduction directly affects energy consumption, operational efficiency, and maintenance requirements. Polymer matrix composites (PMCs) have therefore become attractive alternatives to conventional metallic materials due to their high specific stiffness and strength, corrosion resistance, and design flexibility. These advantages have motivated their widespread application in aerospace, automotive, marine, and mechanical systems, where reductions in structural mass translate into improved system-level performance [1,2,3].

**Figure 1:**
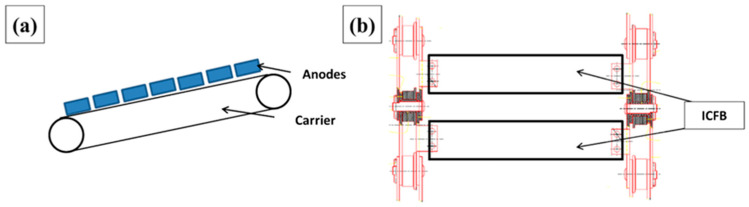
(**a**) Schematic drawing of a slat-type current conveyor for transporting anodes. (**b**) Schematic top view of two industrial conveyor flight bars (ICFBs) mounted on the chain.

In industrial conveyor systems, mass reduction represents a particularly relevant design objective. Long-pitch chain conveyors commonly employ steel industrial conveyor flight bars (ICFBs)—also known as taliscas—as load-bearing elements responsible for supporting and transporting heavy components, such as anodes in aluminum production plants (Figure 1). These flight bars can account for nearly 50% of the total conveyor mass, significantly influencing power demand, energy consumption, and wear of mechanical components. Reducing the mass of flight bars without compromising structural integrity therefore represents both an engineering challenge and an opportunity to improve the energetic and operational efficiency of conveyor systems [4,5].

Fiber-reinforced polymer composites constitute promising candidates for this application. Synthetic fibers such as E-glass, carbon, and aramid (Kevlar) offer distinct combinations of stiffness, strength, density, and cost, enabling tailored structural performance through appropriate material selection and laminate design. Woven fabric architectures, in particular, provide balanced in-plane properties, improved manufacturability, and reduced sensitivity to handling damage compared to unidirectional laminates. Despite these advantages, the application of fiber-reinforced composites as load-bearing elements in industrial conveyors remains relatively underexplored in the literature [6,7,8,9,10].

Existing studies on laminated composites have predominantly focused on aerospace and automotive components, often addressing flat plates, beams, or generic structural elements subjected to idealized loading conditions. While these investigations have established robust theoretical and experimental frameworks for understanding laminate behavior, they provide limited insight into the structural response of conveyor components characterized by real industrial geometry, service loads, and system-level constraints. In particular, few studies explicitly evaluate ply-level stress distributions in composite conveyor elements subjected to operational bending moments derived from industrial conditions, or relate these stresses to energy-related performance indicators [11,12,13,14].

Another limitation of existing work lies in the weak integration between materials science and system-level engineering considerations. Although numerous studies report elastic properties and failure behavior of composite laminates, fewer investigations understand how fiber morphology, lamina anisotropy, and laminate stiffness influence not only structural safety but also broader system metrics such as mass reduction, power demand, and energy consumption. As a result, the literature lacks a coherent framework linking microstructural characteristics of reinforcing fibers to laminate-level stress distributions and, ultimately, to operational performance in heavy-duty industrial systems [15,16,17].

In this context, the present study investigates epoxy matrix composites reinforced with glass, carbon, and Kevlar fibers as potential replacements for conventional steel flight bars in an industrial conveyor system. A multi-scale analytical framework combining micromechanical modeling, Classical Laminate Theory (CLT), and ply-level failure criteria is employed to evaluate the structural response of composite flight bars subjected to a real industrial bending moment. Experimental tensile testing of a vacuum-infused woven glass/epoxy laminate is used to validate micromechanical assumptions and calibrate elastic properties, while carbon/epoxy and Kevlar/epoxy systems are assessed using established handbook data.

The objective of this work is to provide a detailed ply-wise structural assessment of orthotropic laminated flight bars and to relate lamina-level stress distributions to system-level performance indicators, including mass reduction and energy consumption. Rather than proposing deterministic design prescriptions, the study aims to demonstrate structural feasibility and energetic potential, while acknowledging that final design decisions—such as motor selection—remain subject to conventional industrial criteria. By explicitly linking micromechanics, laminate theory, and system-level analysis, this work contributes to the understanding of structure–property–performance relationships in polymer-matrix composites applied to industrial conveyor systems.

## 2. Materials and Methods

### 2.1. Case-Study Conveyor System

The structural design and analysis were carried out using a real industrial conveyor as a case study. The system consists of a long-pitch chain conveyor equipped with flight bars (taliscas), used for transporting anodes in an aluminum production plant. The conveyor has a total length of 32,000 mm, a width of 980 mm, an inlet elevation of 1600 mm and an outlet elevation of 1000 mm, operating at a nominal speed of 6 m/min and designed to convey 19 anodes of 1080 kg each in a single batch.

The conveyor chain is composed of two parallel lines of links and multiple mechanical elements, including external and internal plates (with and without seats), locking bars, bushings, pins, plastic rollers and flight bars. For the 32 m conveyor considered, the assembly comprises 422 flight bars distributed along the two chain lines.

**Figure 2:**
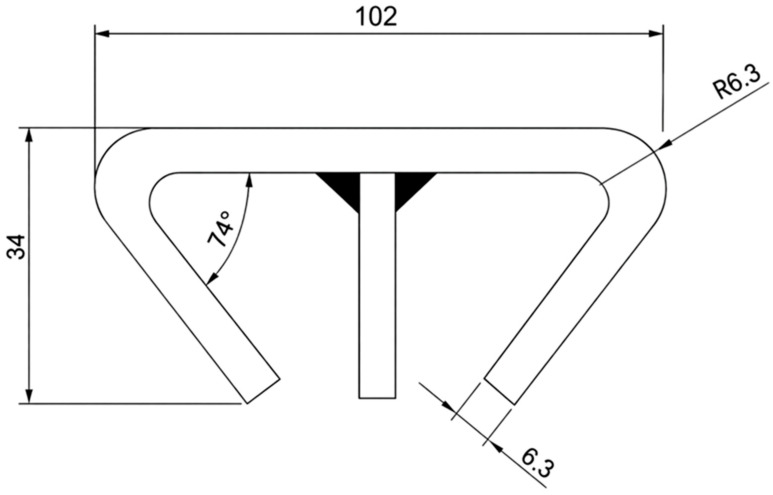
Schematic drawing of a steel slat.

A mass inventory of the chain components shows that the total mass of the conveyor is 9529.62 kg, of which 4.64 t correspond to the flight bars alone (48.7% of the total mass). This makes the flight bars a strategic component for weight reduction and, consequently, for improving the energy efficiency of the drive system. Figure 2 illustrates the steel flight bar geometry adopted as the baseline reference for all comparative analyses.

### 2.2. Baseline Steel Flight-Bar Design

The reference configuration uses flight bars manufactured from SAE 1020 carbon steel, which in this study is taken to have an admissible tensile stress of 68.6 MPa. Each bar spans 960 mm between chain supports and is subjected to bending caused by the distributed weight of the transported anodes. Considering the conveyor capacity of 641.25 kg/m (6290.66 N/m) and the fact that each anode is supported by eight flight bars, the design load per bar is 102.15 kg (1002.10 N). Under these conditions, the bending moment acting at mid-span reaches 342.02 N·m (342,020 N·mm). The steel bar possesses a section modulus of 10,882 mm^3^, and the corresponding flexural stress is therefore 31.44 MPa, obtained directly from the ratio between the applied bending moment and the section modulus. This stress level represents approximately 45.8% of the admissible tensile limit of the material, indicating that the steel flight bar operates with an adequate safety margin under static loading. Shear stresses remain comparatively small and do not govern the structural response. These values establish a consistent mechanical baseline for evaluating the composite alternatives developed in the subsequent sections, ensuring that all material substitutions are assessed under identical loading and geometric conditions.

### 2.3. Composite Materials and Laminate Configuration

To investigate lightweight alternatives to the baseline SAE 1020 steel flight-bar design, three polymer matrix composite systems were considered: glass/epoxy, carbon/epoxy, and Kevlar (aramid)/epoxy. These reinforcement systems were selected because they represent the most widely used classes of synthetic fibers in structural composite applications, covering a broad range of stiffness, strength, density, and industrial maturity. This selection enables a systematic comparison between materials with distinct mechanical efficiencies while maintaining relevance to large-scale engineering applications.

For all composite configurations, bidirectional woven fabrics with a nominal 0°/90° architecture were adopted. This choice reflects common industrial practice, ensures balanced in-plane behavior, and avoids coupling effects associated with highly anisotropic or unsymmetric layups. By maintaining an identical laminate architecture for all materials, differences in structural response are attributed primarily to the intrinsic mechanical characteristics of the reinforcing fibers rather than to geometric or stacking-sequence variations.

Two distinct glass/epoxy laminates are referenced in this study, each serving a different purpose within the multi-scale analysis framework. The first is a conceptual calibration laminate, defined exclusively for micromechanical consistency and lamina-property definition. This laminate consists of ten woven plies with an average ply thickness of approximately 0.32 mm, resulting in a nominal total thickness of about 3.20 mm. It is not structurally tested and does not represent the actual thickness of the flight bar. Instead, it is introduced to establish representative ply thickness, fiber volume fraction assumptions, and lamina-level elastic properties used as input for the Classical Laminate Theory (CLT) formulation.

The second laminate is an experimental validation laminate, manufactured specifically for tensile testing and described in detail in Section 2.7. This laminate consists of five bidirectional woven plies, resulting in a total thickness of approximately 1.6 mm, and was produced via the Vacuum Infusion Process (VIP). Its sole purpose is to provide experimental validation of the elastic modulus and tensile strength of the woven glass/epoxy system, thereby supporting the micromechanical assumptions adopted in the analytical model. This experimental laminate is not intended to represent either the conceptual calibration laminate or the structural thickness of the flight bar.

For the carbon/epoxy and Kevlar/epoxy systems, no experimental laminates were fabricated within the scope of this study. Their lamina-level elastic properties were therefore obtained from established composite materials handbooks and datasheets, following standard practice in preliminary composite structural design. This approach enables a consistent comparative assessment across different reinforcement systems without requiring a full experimental campaign for each material.

It is emphasized that the experimental validation performed in this study was intentionally limited to the glass/epoxy system. This material was selected as a validation anchor because it represents the mechanically most critical configuration among the investigated composites, exhibiting the lowest stiffness and the highest predicted stress levels under the same bending moment. Consequently, experimental confirmation of the glass/epoxy response provides a conservative benchmark for assessing the reliability of the analytical framework applied to the stiffer carbon/epoxy and Kevlar/epoxy systems.

For structural evaluation purposes, the flight bar itself was idealized as a fully laminated solid composite section occupying the entire external geometry of 60 mm × 20 mm, identical to the steel reference component. The composite flight bar was therefore modeled as an equivalent symmetric cross-ply laminate of the form [0/90]^N^, where N is sufficiently large to reproduce the full section height. This equivalent-laminate representation enables the application of Classical Laminate Theory in a manner fully consistent with the bending lever arm, laminate curvature, bending stiffness matrix [D], mass calculation, and system-level energy analysis.

The elastic properties adopted as input for the CLT formulation correspond to lamina-level, unidirectional-equivalent constants, suitable for representing woven laminates through standard homogenization practice. For the glass/epoxy system, two sets of elastic constants were considered: (i) typical handbook values representative of higher fiber volume fractions, and (ii) calibrated properties derived from experimental tensile testing of the woven laminate. For the carbon/epoxy and Kevlar/epoxy systems, lamina properties were taken directly from literature sources. These elastic constants are summarized in Table 1.

By explicitly distinguishing between the conceptual calibration laminate, the experimental validation laminate, and the equivalent structural laminate used in the flight-bar analysis, this modeling strategy ensures full internal consistency between micromechanics, laminate theory, geometric assumptions, mass estimation, and energy-consumption evaluation. This clarification eliminates ambiguity regarding laminate thickness, number of plies, and experimental scope, and reinforces the robustness of the multi-scale framework adopted in this study.

The elastic constants listed in Table 1 are independent of laminate thickness and are used solely to construct the reduced stiffness matrices of the laminae within the CLT framework. These properties define the orthotropic response of each ply and, when integrated through the full laminate thickness, govern the bending stiffness and ply-level stress distribution of the equivalent composite flight bar.

By explicitly separating the experimentally characterized laminate used for property calibration from the equivalent structural laminate used for the flight-bar analysis, this modeling strategy ensures internal consistency between micromechanics, laminate theory, geometric assumptions, mass estimation, and energy-consumption evaluation. The geometric and sectional parameters adopted for the composite flight-bar structural model are detailed in Section Geometry and Sectional Properties of the Composite Flight Bar and summarized in Table 2, while the laminate stiffness formulation and ply-level stress evaluation are presented in Section 2.5.

#### Geometry and Sectional Properties of the Composite Flight Bar

To enable a direct and rigorous comparison between the conventional SAE 1020 steel flight bar and the proposed composite alternatives, all designs were analyzed using the same external geometric envelope adopted in the industrial conveyor system. The flight bar has a rectangular cross section with a width of 60 mm and a height of 20 mm, and these dimensions were preserved for all materials to ensure geometric compatibility with the chain supports and to isolate the effect of material substitution on the structural response.

Although the external geometric envelope of the composite flight bar is identical to that of the steel reference component (60 × 20 mm^2^), the structural model adopts a fully solid rectangular composite section occupying the entire cross-section. This representation does not imply that the component is manufactured as a monolithic laminate in a single processing step. Instead, it constitutes an equivalent structural idealization introduced to ensure geometric compatibility, mass consistency, and energetic equivalence with the steel reference configuration, while enabling the direct application of Classical Laminate Theory. By assuming the entire cross-section as load-bearing composite material, the analysis remains conservative and avoids artificially increasing bending stiffness or underestimating stresses.

Although glass/epoxy laminates with nominal thicknesses of approximately 3.20 mm (ten plies) and 1.6 mm (five plies) are referenced elsewhere in this study, it is emphasized that neither laminate represents the structural thickness of the flight bar. The ten-ply laminate is introduced exclusively as a conceptual calibration laminate for micromechanical consistency and lamina-property definition, while the five-ply laminate corresponds to the experimental validation laminate manufactured for tensile testing, as described in Section 2.7. Both laminates serve auxiliary roles within the multi-scale modeling framework and are not intended to represent the load-bearing configuration of the flight bar.

For structural evaluation purposes, the composite flight bar is idealized as an equivalent fully laminated composite section occupying the entire 60 mm × 20 mm cross section, analogous to the steel reference component. The section is modeled as a symmetric cross-ply laminate of the form [0/90]^N^, where the total laminate thickness is equal to the full section height of 20 mm. In this representation, the laminate mid-plane coincides with the geometric mid-plane of the flight bar, and the thickness coordinate z is defined over the interval −10 mm ≤ z ≤ +10 mm.

The laminate formulation adopted does not imply the absence of ply discretization. Instead, it represents the converged limit of a finely discretized laminated section, in which the number of plies N is sufficiently large and the ply thickness sufficiently small such that further subdivision of the thickness does not affect the bending stiffness matrix [D] nor the resulting ply-level stress distributions. In this converged regime, the laminate response depends only on the lamina elastic properties and the total thickness, and not on the specific number of plies used in the discretization. Consequently, the structural behavior of the flight bar can be reproduced by discretizing the 20 mm thickness using any sufficiently small ply thickness.

A reference ply thickness of approximately 0.32 mm, obtained from the conceptual calibration laminate, is used solely to define representative lamina properties and to maintain micromechanical consistency. It does not imply that the structural laminate consists of a discrete stacking sequence identical to the calibration laminate. Instead, the equivalent-laminate approach provides a homogenized representation suitable for evaluating bending stiffness and ply-level stresses under service loading.

The bending moment applied to the composite flight bar is identical to that acting on the steel reference component, namely 342.02 N·m, ensuring energetic equivalence across all material systems. In the CLT framework, this moment is treated as a bending moment resultant per unit width, consistent with the plate-based formulation adopted in this study. The maximum distance to the outer fibers is therefore z_max_ = ±10 mm, corresponding to the full section height.

The moment of inertia of the steel reference section, derived from the section modulus reported in Section 2.2, is given by Equation (1):

(1) $$I_{steel}=W_{steel}\times \frac{h}{2}=10.882\;{mm}^{3}\times 10\;mm=108.820\;{mm}^{4}$$

This value is used solely as a benchmark for comparison. In the composite configurations, bending stiffness and stresses are obtained directly from the laminate bending stiffness matrix [D], without invoking an equivalent isotropic moment of inertia. This distinction preserves the anisotropic nature of the composite materials and avoids artificial equivalence assumptions.

Finally, it is emphasized that the mass of the composite flight bars—and the corresponding energy-consumption analysis presented in Section 2.8—was calculated assuming that the entire 60 mm × 20 mm cross section is composed of composite material. No low-density core, metallic insert, or non-structural filler was considered in the present study. This assumption ensures consistency between the structural model, mass estimation, and system-level energy analysis, and represents a conservative scenario for industrial implementation.

It is important to clarify that the [0/90]^N^ laminate adopted in this study represents an equivalent structural idealization rather than a literal manufacturing layup produced in a single processing step. The assumption of a fully solid 20 mm thick laminated section is introduced exclusively for structural analysis purposes, allowing the application of Classical Laminate Theory in a converged discretization regime and enabling direct comparison with the solid steel reference geometry.

In practical industrial manufacturing, a composite component of this thickness would not be produced as a single monolithic laminate via one-step vacuum infusion. Instead, established composite manufacturing routes—such as multi-stage vacuum infusion, sub-laminate co-curing, secondary bonding, prepreg layup with intermediate debulking, resin transfer molding (RTM), or pultrusion—are commonly employed to fabricate thick composite sections while mitigating risks of void formation, resin accumulation, and interlaminar defects.

Potential manufacturing-induced phenomena such as delamination, resin-rich regions, voids, and thickness-dependent consolidation effects are therefore recognized as relevant to real components of this scale. However, these effects are beyond the scope of the present analytical feasibility study, which focuses on elastic structural response and ply-level stress distributions under service bending loads. Their influence on damage tolerance and long-term durability should be addressed through dedicated manufacturing trials and experimental characterization in future work.

Table 2 summarizes the geometric and sectional parameters adopted for the composite flight-bar analysis.

### 2.4. Micromechanical Modelling of Lamina Properties

Since Classical Laminate Theory (CLT) requires lamina-level orthotropic elastic properties as input, and the mechanical constants of bidirectional woven fabrics are not directly available in classical composite handbooks, the woven plies were modeled using equivalent unidirectional (UD) lamina properties. This homogenization strategy assumes that each woven ply can be represented by two orthogonal families of fiber tows (warp and weft), each contributing to the in-plane stiffness according to its orientation. As a result, the woven ply is treated as an equivalent orthotropic lamina suitable for use within the CLT framework.

In this approach, the longitudinal elastic modulus of the woven ply along the 0° direction is approximated by the effective UD longitudinal modulus of the fiber–matrix system, while the transverse modulus along the 90° direction is represented by the corresponding UD transverse modulus. Similarly, the in-plane shear modulus and Poisson’s ratio are taken from UD-equivalent lamina data and interpreted as effective orthotropic constants of the woven ply. This procedure neglects mesoscale effects such as tow crimp, fiber undulation, nesting, and resin-rich regions, but provides a first-order approximation of the elastic response that is widely accepted for global stiffness and stress predictions in laminated structures subjected predominantly to bending loads.

This homogenization approach—treating the woven ply as an equivalent orthotropic lamina (or, equivalently, as a cross-ply representation at the yarn/tow level) and subsequently applying CLT/ABD formulations at the laminate scale—is consistent with current multiscale modeling practice for woven composites. Recent studies have shown that yarn- or tow-level homogenization followed by laminate-level analysis using CLT provides accurate predictions of laminate stiffness and stress distributions when compared with detailed mesoscale or finite element models, particularly for thin and bending-dominated structures [6,21,22].

The lamina properties were determined using classical micromechanical relations and the Rule of Mixtures, consistent with the theoretical framework presented in the study for orthotropic laminae. For a unidirectional lamina, the longitudinal elastic modulus E_L_ was obtained as Equation (2):

(2) $$E_{L}=E_{f}V_{f}+E_{m}V_{m}$$

where E_f_ and E_m_ are the fiber and matrix moduli, respectively, and V_f_ and V_m_ are the corresponding volume fractions. The transverse modulus E_T_ was estimated using an inverse Rule of Mixtures as Equation (3):

(3) $$\frac{1}{E_{T}}=\frac{V_{f}}{E_{f}}+\frac{V_{m}}{E_{m}}$$

Similarly, the in-plane shear modulus G_LT_ and Poisson’s ratio v_LT_ were obtained from standard micromechanical expressions for continuous, perfectly bonded fibers in an isotropic matrix. These lamina-level properties were later used to construct the stiffness matrices of the laminates according to Classical Laminate Theory (Section 2.5).

For the glass/epoxy system, the micromechanical estimates were additionally evaluated using the experimentally inferred V_f_ (≈0.27). This produces a longitudinal modulus in the range of 22–26 GPa, consistent with the measured tensile modulus (25.0 ± 2.5 GPa), thereby reconciling the difference between handbook UD-equivalent values and the infused woven laminate response.

### 2.5. Laminate Analysis by Classical Laminate Theory

**Figure 3:**
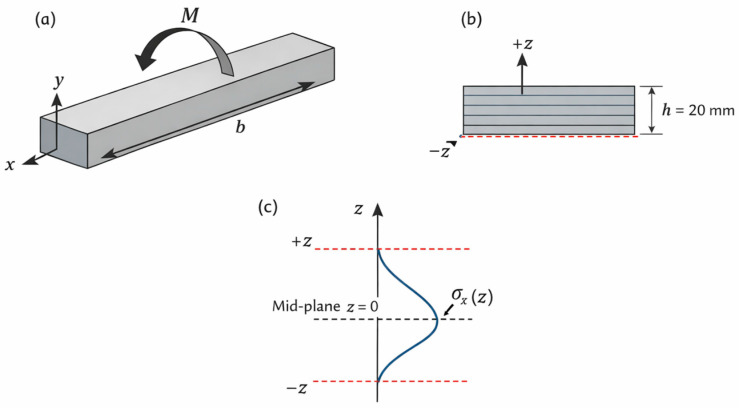
Schematic representation of the composite flight bar and the Classical Laminate Theory (CLT) framework adopted: (**a**) flight-bar geometry, coordinate system, and applied bending moment M; (**b**) laminated cross-section showing the total thickness (h = 20 mm), laminate mid-plane (z = 0), and thickness coordinate z; (**c**) qualitative longitudinal stress distribution σ_x_(z) through the laminate thickness under bending.

The structural behavior of the composite flight bars was analyzed using Classical Laminate Theory (CLT), following the standard macromechanical formulation for orthotropic laminae and multilayered composites. In the present study, CLT is employed to evaluate the bending response and ply-level stress distribution of an equivalent laminated composite section representing the full 60 mm × 20 mm cross section of the flight bar. Figure 3 illustrates the geometry, coordinate system, bending configuration, and stress distribution adopted for the CLT-based analysis.

The application of CLT is based on the classical assumptions of laminated plate theory: (i) each lamina behaves as a homogeneous, linearly elastic orthotropic material in its principal material directions; (ii) perfect bonding exists between adjacent plies, ensuring strain compatibility across interfaces; (iii) transverse normals remain straight and perpendicular to the laminate mid-plane during deformation (Kirchhoff–Love hypothesis); (iv) transverse normal stresses and interlaminar shear stresses are negligible compared with in-plane stress components; and (v) the laminate is symmetric with respect to its mid-plane, such that bending–extension coupling is absent and the coupling matrix [B] is identically zero. These assumptions are appropriate for the present application, given the slender geometry of the flight bar and the predominance of bending loads under service conditions.

For structural modeling purposes, the composite flight bar is idealized as a symmetric cross-ply laminate of the form [0/90]^N^, occupying the entire section height of 20 mm. Although Classical Laminate Theory is traditionally formulated for explicitly discretized laminates, the present analysis adopts an equivalent finely discretized laminate representation. In this formulation, the number of plies N is assumed to be sufficiently large, and the ply thickness sufficiently small, such that further subdivision of the laminate thickness does not modify the bending stiffness matrix [D] nor the resulting ply-level stress distributions. In this converged regime, the laminate response depends only on the lamina elastic properties and the total laminate thickness, and not on the specific number of plies used in the discretization.

Mathematically, this approach corresponds to the limit of Classical Laminate Theory for an infinitely refined laminate. The bending stiffness matrix [D] is obtained by integrating the transformed reduced stiffness matrices of the laminae through the full thickness of the section, defined over the interval −h/2 ≤ z ≤ +h/2, where h = 20 mm. This formulation is fully reproducible, since identical results are obtained by discretizing the laminate using any sufficiently small ply thickness. In practical terms, discretizations using ply thicknesses on the order of 0.25–0.32 mm already yield converged values of [D] and ply-level stresses, confirming the independence of the solution from the chosen discretization once convergence is achieved.

For each lamina, the reduced stiffness matrix [Q] was constructed using the lamina-level elastic constants listed in Table 1. These constants correspond to unidirectional-equivalent orthotropic properties and are independent of the laminate thickness. For plies oriented at 0° and 90°, the transformed reduced stiffness matrices $\left[\bar{Q}\right]$ were obtained using standard tensor transformation relations. The extensional [A], coupling [B], and bending [D] stiffness matrices of the equivalent laminate were computed as Equation (4):

(4) $$A=\sum_{k}{\bar{Q}}_{k}\left(z_{k}-z_{k-1}\right),\;B=\frac{1}{2}\sum_{k}{\bar{Q}}_{k}\left({z^{2}}_{k}-{z^{2}}_{k-1}\right),D=\frac{1}{3}\sum_{k}{\bar{Q}}_{k}({z^{3}}_{k}-{z^{3}}_{k-1})$$

where z_k_ and z_k−1_ denote the top and bottom coordinates of the k-th ply measured from the laminate mid-plane. Owing to the symmetric cross-ply stacking sequence adopted, the coupling matrix [B] vanishes, simplifying the constitutive relations.

The operational bending moment acting on the steel flight bar, (M = 342.02 N·m), was adopted as the design bending load for all composite configurations. In the CLT framework, this moment was converted into a bending moment resultant per unit width Equation (5):

(5) $$M_{x}=\frac{M}{b}$$

where b = 60 mm is the flight-bar width. This conversion ensures energetic equivalence between the beam representation of the flight bar and the plate-based CLT formulation and is standard practice in the analysis of laminated beams with constant width.

Since the flight bar is subjected exclusively to bending and no in-plane membrane loads are applied, the mid-plane strain vector is zero. The laminate curvatures are therefore obtained directly from Equation (6):

(6) $$\left\{k\right\}={\left[D\right]}^{-1}\left\{M\right\}$$

where $\left\{M\right\}={\left\{M_{x},0,0\right\}}^{T}$. The strain field at an arbitrary distance z from the laminate mid-plane is then given by Equation (7):

(7) {ε(z)}=z{k}

For each ply, the strain vector evaluated at the ply mid-surface was transformed into the local material coordinates, and the corresponding stress components were computed as Equation (8):

(8) $${\left\{\sigma \right\}}^{\left(k\right)}={\left[\bar{Q}\right]}_{k}\left\{\varepsilon \left(z_{k}\right)\right\}$$

This procedure yields the ply-level longitudinal, transverse, and in-plane shear stresses used in the subsequent failure analyses based on the Maximum Stress and Azzi–Tsai–Hill criteria.

Although the flight bar is a beam-like component, the CLT-based formulation remains valid due to the high slenderness ratio of the structure, with a span-to-height ratio of approximately L/h ≈ 48. Under these conditions, transverse shear deformation is negligible and the structural response is governed by bending, such that the CLT plate formulation provides results equivalent to laminated Euler–Bernoulli beam theory. Recent studies have confirmed that, in this regime, the laminate bending stiffness derived from the [D] matrix leads to accurate stress and curvature predictions for composite beams under static bending [23,24].

Although Classical Laminate Theory is formally derived for thin laminated plates, its application to the present composite flight bar is justified by the high slenderness ratio of the component. The bar span-to-height ratio is approximately L/h ≈ 48, which places the structure well within the slender beam regime, where transverse shear deformation contributes only marginally to the total deflection and stress distribution.

For laminated beams with L/h ratios above approximately 20–25, classical Euler–Bernoulli assumptions—namely that plane sections remain plane and transverse shear effects are negligible—have been shown to provide accurate predictions of bending stresses and curvatures. Under these conditions, the bending response derived from the laminate bending stiffness matrix [D] is energetically equivalent to that obtained from laminated beam theory, as previously demonstrated in the literature.

Interlaminar stresses and transverse shear stresses, which are not explicitly captured by Classical Laminate Theory, are expected to be localized near supports and ply interfaces and do not govern the global elastic response of the flight bar under static bending. Their potential role in damage initiation or delamination is acknowledged; however, such effects fall outside the scope of the present first-order structural feasibility assessment and would require higher-order theories or three-dimensional finite element modeling for detailed evaluation.

No higher-order laminate theory or Timoshenko-type shear correction was implemented in the present analysis. This choice was intentional and consistent with the objectives of the study, which focuses on first-order elastic stress evaluation under bending-dominated loading conditions.

For the investigated flight bar, with a span-to-height ratio of approximately L/h ≈ 48, transverse shear deformation is expected to contribute primarily to overall deflection rather than to longitudinal stress distribution. Numerous studies on laminated beams have shown that, in this slenderness regime, the inclusion of Timoshenko shear correction or first-order shear deformation theories (FSDT) leads to only marginal changes in bending stresses, while the predicted curvatures and deflections may differ slightly.

Since the failure assessment in this work is based on ply-level normal and in-plane shear stresses obtained under linear elastic bending, the omission of transverse shear deformation does not significantly influence the stress-based failure indices or the structural feasibility conclusions. The potential influence of shear effects becomes relevant for short-span components, thick sandwich structures, or when interlaminar damage and delamination are explicitly modeled, which is beyond the scope of the present study.

It is emphasized that the CLT formulation adopted here evaluates stresses exclusively within the equivalent laminated composite section occupying the full 60 mm × 20 mm cross section. No partial-thickness structural assumptions, non-structural cores, or filler materials are considered. This ensures full consistency between laminate theory, geometric idealization, mass calculation, and system-level energy analysis.

Finally, it should be noted that the present analysis is restricted to linear elastic behavior under static bending loads. Damage initiation, delamination, fatigue, and progressive failure mechanisms are not considered within the CLT framework and are therefore beyond the scope of this initial structural feasibility assessment.

It is important to clarify that the bending moment adopted in this study (342.02 N·m) represents an equivalent design bending moment derived from the static load distribution of the conveyor under nominal operating conditions. This value corresponds to the most critical bending demand acting on the flight bars and is intentionally employed as a reference load for a first-order structural feasibility assessment.

Although industrial conveyor flight bars are subjected in service to cyclic loading, localized impacts during material transfer, and combined stress states involving bending, torsion, and shear, these effects were not explicitly modeled in the present work. Instead, the analysis focuses on the dominant bending response, which governs the global stress state due to the high slenderness ratio of the flight bar and the load-support configuration of the conveyor system.

The adopted approach is consistent with preliminary composite structural design practices, in which elastic stress levels under representative worst-case bending loads are first verified before more advanced analyses—such as fatigue, impact, and multi-axial loading—are conducted.

The structural integrity of the composite flight bars was evaluated at the ply level using stress-based failure criteria derived from Classical Laminate Theory. In the present study, two well-established criteria were adopted: the Maximum Stress criterion and the Azzi–Tsai–Hill (ATH) quadratic interaction criterion.

The Maximum Stress criterion was applied to all laminate configurations (glass/epoxy, carbon/epoxy, and Kevlar/epoxy) due to its simplicity, transparency, and widespread use in preliminary composite structural design. This criterion allows direct comparison between individual stress components and their corresponding lamina strength limits, enabling a clear identification of dominant stress modes.

The Azzi–Tsai–Hill criterion was additionally applied to the glass/epoxy laminate, for which experimental tensile data were available. The ATH criterion accounts for the interaction between longitudinal, transverse, and in-plane shear stresses and therefore provides a more comprehensive assessment of multidimensional stress states. Its application was intentionally restricted to the glass/epoxy system to avoid extrapolating interaction-based failure models to material systems not experimentally validated within the scope of this study.

More advanced failure models, such as Hashin-, Puck-, or LaRC-type criteria, were not employed, as the present work focuses on first-order elastic feasibility rather than progressive damage or failure mode discrimination.

It is emphasized that the objective of the present analysis is not to provide full experimental or numerical validation of the structural response of the composite flight bars, but rather to establish a first-order structural feasibility assessment under representative service bending loads. Classical Laminate Theory combined with stress-based failure criteria is widely adopted in preliminary composite structural design to verify elastic stress levels prior to more advanced analyses. Experimental flexural testing or three-dimensional finite element simulations were therefore not included at this stage and are identified as necessary steps for detailed validation and industrial qualification in future work.

#### 2.5.1. Lamina Strength Properties Used in the Failure Criteria

The application of the Maximum Stress and Azzi–Tsai–Hill failure criteria require the lamina strength parameters in the principal material directions, namely the longitudinal tensile and compressive strengths (Xt, Xc), the transverse tensile and compressive strengths (Yt, Yc), and the in-plane shear strength (S). These properties were not directly measured in the present study; therefore, well-established values from composite materials handbooks were used to ensure consistency and reproducibility. The selected values are representative of woven bidirectional laminates and have been widely adopted in analytical studies based on Classical Laminate Theory.

Table 3 summarizes the strength parameters adopted for the glass/epoxy, carbon/epoxy, and Kevlar/epoxy systems, along with their respective sources. These values were used uniformly across all plies of each laminate to compute the Maximum Stress and Azzi–Tsai–Hill indices in Section 3.1, Section 3.2 and Section 3.3. The use of documented handbook values ensures that the failure assessment is fully traceable and reproducible by other researchers.

The strength properties reported in Table 3 correspond to typical unidirectional (UD) lamina values commonly adopted in Classical Laminate Theory–based failure assessments. Their use in the present study does not imply that the woven bidirectional laminates exhibit identical strength levels to UD laminates. Instead, these values are employed as reference strength limits to enable a conservative and standardized comparison of ply-level stress states using stress-based failure criteria.

It is well established that woven fabrics generally exhibit lower tensile and compressive strengths than UD laminas due to fiber crimp, tow undulation, and resin-rich regions. Consequently, the use of UD lamina strength data may lead to an overestimation of the actual strength of woven laminates if interpreted as admissible design values. For this reason, the present work does not treat the UD strength values as allowable stresses for the woven systems, but rather as benchmark limits within a first-order feasibility assessment.

For the glass/epoxy system, this potential limitation is explicitly addressed by additionally comparing the predicted service stresses with the experimentally measured tensile strength of the woven laminate, thereby providing a direct and conservative validation of structural feasibility. For the carbon/epoxy and Kevlar/epoxy systems, the strength values are used solely for comparative assessment under identical assumptions, and the results should be interpreted as preliminary indicators rather than definitive design allowable.

#### 2.5.2. Consistency Check with Laminated Beam Theory

To ensure that the plate-based CLT formulation does not distort the structural response of the flight bar, a conceptual consistency check can be established with classical laminated beam theory. For a slender laminated beam subjected to pure bending, the curvature κ_x_ is related to the applied bending moment by Equation (9):

(9) $$k_{x}=\frac{M}{E_{eq}I_{eq}}$$

where E_eq_ and I_eq_ represent the equivalent bending modulus and moment of inertia of the laminate section. In CLT, the same curvature is obtained from Equation (10):

(10) $$k_{x}={D_{11}}^{-1}M_{x}$$

With Mx = M/b. Since D_11_ = E_eq_I_eq_/b, both formulations are energetically and kinematically equivalent. Therefore, the CLT-based plate formulation employed in this study provides a consistent representation of the bending response of the composite flight bar and yields ply-level stresses equivalent to those predicted by laminated Euler–Bernoulli beam theory under the same loading conditions.

### 2.6. Design Limit According to ABNT NBR 14574

The Brazilian standard ABNT NBR 14574 [25] proposes, for preliminary structural design of polymer-matrix composite members, a conservative working-stress reference defined as 30% of the longitudinal tensile strength of a unidirectional (UD) lamina. This recommendation is intended as a normative benchmark for UD-based allowable and provides an initial reference level to account for uncertainties associated with material variability, manufacturing defects, and service conditions.

In the present study, this 30% criterion is used only as a UD-based normative benchmark and is not treated as the admissible stress of the woven laminate tested experimentally. This distinction is necessary because the flight-bar laminates investigated here are based on bidirectional woven fabrics (0°/90°), whose tensile strength may be substantially lower than the UD lamina strength due to crimp, tow undulation, and resin-rich regions. Therefore, UD-based allowable are not directly transferable to the woven laminate without additional qualification.

For the glass/epoxy system, the handbook UD lamina strength ${X_{t}}^{UD}$ = 1062 MPa yields a UD-based benchmark of 0.30 ${X_{t}}^{UD}$ = 318.6 MPa. However, the experimentally measured ultimate tensile strength of the vacuum-infused woven glass/epoxy laminate is σ_u,exp_ ≈ 280 MPa (Section 3.1), which provides the most relevant strength reference for the woven architecture investigated. Accordingly, the structural feasibility of the glass/epoxy flight bar is assessed against the experimental laminate strength by verifying that the maximum service stress satisfies σ_x,max_ < σ_u,exp_, and the resulting margin of safety is reported in the Results section.

For carbon/epoxy and Kevlar/epoxy systems, experimental laminate strength data were not generated in this study; therefore, strength references were taken from established handbooks and datasheets for comparative assessment. In all cases, the stress-based results should be interpreted as a first-order feasibility evaluation under static bending, not as a full normative certification.

### 2.7. Fabrication and Tensile Testing of the Glass/Epoxy Laminate

**Figure 4:**
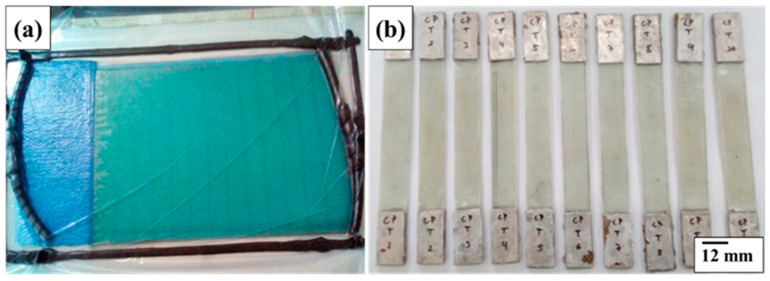
(**a**) Composite board immediately after epoxy resin infusion. (**b**) Test specimens generated from the plate.

To validate the analytical estimates adopted for the glass/epoxy system, an experimental tensile characterization was performed on a laminate manufactured via the Vacuum Infusion Process (VIP). This experimentally manufactured laminate is distinct from the conceptual ten-ply laminate referenced in Section 2.3 and Section Geometry and Sectional Properties of the Composite Flight Bar and consists of five bidirectional woven plies, resulting in a total laminate thickness of approximately 1.6 mm. Figure 4 shows the vacuum-infused glass/epoxy laminate plate and presents the ASTM D3039 [26] tensile specimens extracted from this plate.

The laminate plate had nominal dimensions of 350 mm in width, 460 mm in length, and 1.6 mm in thickness. It was composed of five layers of bidirectional E-glass fabric (0°/90°) with an areal weight of approximately 330 g/m^2^, resulting in a total glass fiber mass of 257 g. The epoxy resin mass used during infusion was 314.11 g, corresponding to approximately 45 wt.% glass fiber and 55 wt.% epoxy matrix in the final laminate.

The epoxy resin system was mixed with its curing agent according to the manufacturer’s technical datasheet, using a mass ratio of 50% curing agent relative to the resin mass. This proportion corresponds to the stoichiometric formulation recommended for the specific epoxy system employed and was adopted to ensure adequate crosslinking and complete curing of the polymer matrix.

After resin infusion, the laminate was cured following the curing schedule recommended by the resin manufacturer. The initial curing stage was carried out at room temperature (23 ± 2 °C) for 24 h under vacuum, allowing complete gelation and primary crosslinking of the epoxy matrix. Subsequently, a post-curing stage was performed in an air-circulating oven at 60 °C for 4 h to promote further crosslinking and stabilize the thermosetting network. After post-curing, the laminate was allowed to cool naturally to room temperature before specimen machining. This curing regimen was selected to ensure adequate polymerization while avoiding excessive thermal gradients, residual stresses, or matrix degradation.

The laminate was manufactured using the Vacuum Infusion Process under a vacuum condition close to full vacuum. During infusion, the pressure in the vacuum line was reduced to approximately −90 kPa (gauge), corresponding to an absolute pressure on the order of 10–15 kPa, in order to promote efficient resin flow, fiber wet-out, and void minimization. The applied vacuum level was maintained throughout the infusion and curing stages, in accordance with standard VIP practice for woven glass/epoxy laminates. Minor fluctuations in vacuum level may occur during infusion due to resin flow dynamics and hose permeability; however, the vacuum remained within the typical range reported for vacuum-infused polymer composites.

Due to the intrinsic characteristics of the vacuum infusion process, small variations in fiber volume fraction, local compaction, resin distribution, and laminate thickness are expected. Typical deviations for VIP laminates include ±3–5% variation in fiber volume fraction and 1–3% internal porosity, as well as local thickness variations on the order of ±0.05–0.10 mm. These sources of variability were considered when interpreting the experimental mechanical results and when reconciling them with the micromechanical predictions presented in Section 2.4 and Section 3.2.

**Figure 5:**
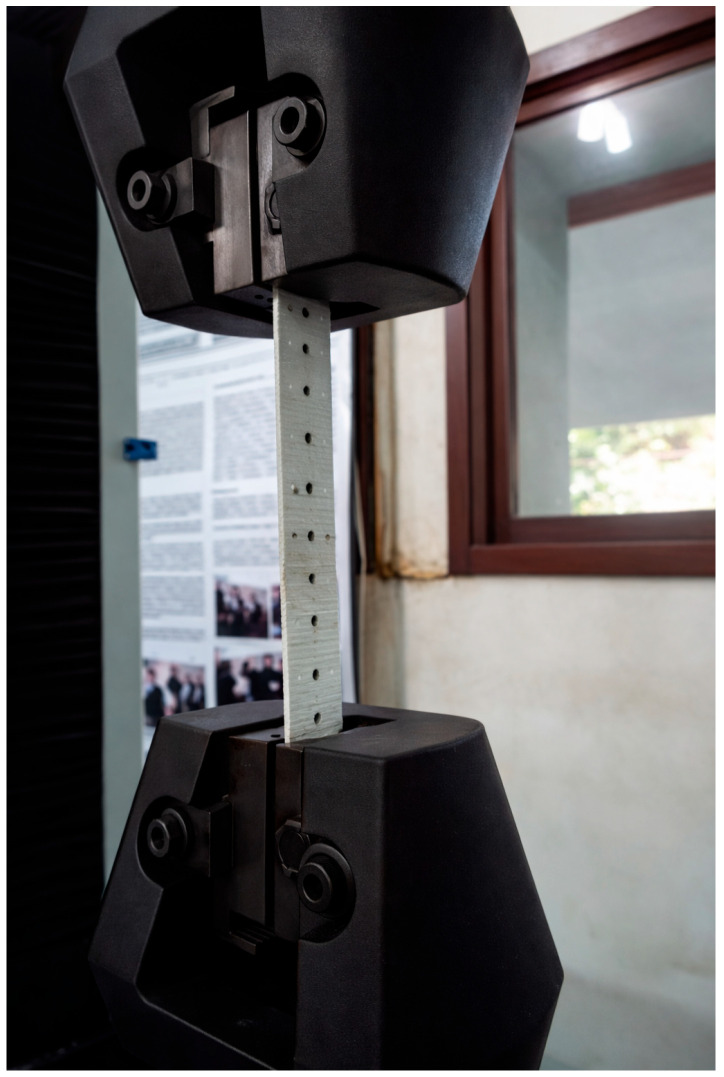
Tensile test setup used for the experimental validation of the glass/epoxy laminate according to ASTM D3039 [26].

From the infused plate, eight tensile specimens were machined and prepared in accordance with ASTM D3039 [26] for polymer matrix composite materials. Tensile tests were conducted using an Arotec WDW-100E universal testing machine (Hongtuo Instrument Co., Ltd., Dongguan, China) at a constant crosshead speed of 5 mm/min, in the metallography laboratory of the same institution. The tests were performed under controlled laboratory conditions, with a temperature of 23 ± 2 °C and relative humidity of 50 ± 5%, consistent with ASTM recommendations. Figure 5 shows the tensile test setup used for the experimental validation of the glass/epoxy laminate, conducted in accordance with ASTM D3039 [26].

The tensile modulus, ultimate tensile strength, and failure strain obtained from these tests were used to validate the micromechanical assumptions adopted for the glass/epoxy system and to calibrate the elastic properties employed in the Classical Laminate Theory (CLT) analysis. It is emphasized that the experimental tensile strength of the woven laminate was used exclusively for model validation and consistency assessment, and not to redefine admissible design stresses, in accordance with the interpretation of ABNT NBR 14574 [25] discussed in Section 2.6.

Finally, it should be noted that the experimental validation performed in this study was limited to tensile testing of a single vacuum-infused glass/epoxy laminate. No additional mechanical tests (e.g., flexural, interlaminar shear, fracture, or fatigue tests) were conducted, nor were carbon/epoxy or Kevlar/epoxy laminates fabricated for experimental comparison. Consequently, the properties adopted for these latter systems rely on micromechanical estimates and well-established handbook data. Although this approach is appropriate for a preliminary structural feasibility study, future work should include additional experimental characterization to support long-term durability assessment and industrial implementation.

The experimental program was intentionally restricted to tensile testing of the glass/epoxy laminate. Carbon/epoxy and Kevlar/epoxy laminates were not fabricated within the scope of this study, as the primary objective was not full experimental qualification but rather a comparative structural feasibility assessment based on micromechanics and Classical Laminate Theory. The use of consolidated handbook properties for these materials follows standard practice in preliminary composite structural design and enables a consistent comparison across reinforcement systems.

### 2.8. Energy Consumption and Motor Power Estimation

The influence of flight-bar mass on the energy consumption of the conveyor system was evaluated by recalculating the mechanical power demand for the baseline steel configuration and for the three composite alternatives. In mechanical conveying systems, the steady-state power requirement is directly related to the total moving mass, since reductions in mass lead to lower resistive forces, reduced torque at the drive unit, and, consequently, lower mechanical power demand. This relationship is a well-established principle in the design of energy-efficient conveying and transport systems and has been widely discussed in recent engineering literature [4,5].

In the reference configuration, the total mass of the 422 steel flight bars is 4.64 t. When steel is replaced by composite materials, this value is reduced to 0.82 t for glass/epoxy, 0.74 t for carbon/epoxy, and 0.68 t for Kevlar/epoxy configurations, corresponding to a mass reduction of approximately 82–85%. This reduction significantly affects the total moving mass of the conveyor system and, therefore, the power required for continuous operation.

It is important to clarify that the reported mass reduction of 82–85% refers exclusively to the flight bars themselves and not to the total moving mass of the conveyor system. In the power analysis, the total moving mass was explicitly recalculated for each configuration by summing the mass of the chain components, the transported load, and the flight bars. Only the mass contribution of the flight bars was modified, while all other components and operating conditions were kept identical to the steel reference configuration. Consequently, the reduction in required mechanical power results from a full recalculation of the total moving mass, rather than from a proportional scaling based on the flight-bar mass fraction.

The tangential force applied to the drive sprocket was estimated using standard conveyor design relations as Equation (11):

(11) $$F_{t}=m_{tot}g(f_{r}cos\alpha +sin\alpha )$$

where m_tot_ is the total moving mass (chain + flight bars + load), g = 9.81 m/s^2^, f_r_ = 0.03 is the rolling-friction coefficient, and α ≈ 1.07° is the conveyor inclination (computed from a height difference of 0.60 m over 32 m). The conveyor speed is v = 0.10 m/s (6 m/min). The mechanical power required at the drive shaft under steady-state operation is given by Equation (12):

(12) $$P_{mec}=F_{t}v$$

To account for drivetrain losses, the installed electrical power was estimated by correcting the mechanical power by the overall system efficiency, assumed as (η_tot_ ≈ 0.73), which incorporates motor and gearbox efficiencies as Equation (13):


(13) $$P_{inst}=\frac{P_{mec}}{\eta_{tot}}$$

Using this formulation, the baseline steel configuration yields a calculated steady-state power demand of approximately 5.5 kW, corresponding to a nominal motor class of 7.5 CV. When composite flight bars are considered, the substantial reduction in moving mass leads to a proportional decrease in the calculated mechanical power demand. For all three composite configurations, the resulting steady-state power requirement falls within a lower nominal power range, on the order of 4.0 kW, corresponding to a 5.5 CV motor class.

It is important to emphasize that this reduction in calculated power demand does not imply that motor downsizing is automatic or mandatory in an industrial context. In practical conveyor systems, motor selection is governed not only by steady-state power requirements, but also by starting torque, transient operating conditions, inertial effects, safety factors, gearbox characteristics, standardized motor ratings, and operational margins. The present analysis demonstrates that the use of composite flight bars reduces the mechanical power required for continuous operation and places the system within a lower nominal power class from an energetic standpoint. Whether this reduction translates into the selection of a lower-rated motor or simply results in operation with reduced motor loading depends on standard industrial design practices and project-specific constraints.

To quantify the operational energy implications of this reduced power demand, the annual energy consumption was estimated assuming continuous operation of the conveyor for 8760 h per year and adopting a Brazilian industrial electricity tariff of R$ 0.671 per kWh (ANEEL, 2019). Under these conditions, the annual energy cost for the steel-based configuration is given by Equation (14):

(14) $$C_{steel}=5.5\times 8760\times 0.671=R\$\;32,330.78$$

For the composite configurations, the corresponding annual cost is given by Equation (15):

(15) $$C_{comp}=4.0\times 8760\times 0.671=R\$\;23,518.00$$

Thus, the adoption of composite flight bars leads to an annual energy saving as Equation (16):

(16) $$∆C=C_{steel}-C_{comp}=R\$\;8812.80$$

independently of the specific composite reinforcement employed. This saving reflects the reduced mechanical power required to drive the conveyor under steady-state conditions and represents a direct operational benefit associated with mass reduction.

The estimated annual energy saving of R$ 8812.80 should be interpreted as a reference value obtained under a specific set of operating assumptions. In practical industrial applications, parameters such as operating hours, rolling friction coefficient, and drivetrain efficiency may vary depending on conveyor design, maintenance condition, load distribution, and operating environment.

To assess the robustness of the energy analysis, a qualitative sensitivity evaluation can be established based on typical industrial parameter ranges. For chain conveyor systems, rolling friction coefficients commonly vary between f_r_ ≈ 0.02–0.05, while overall motor–gearbox efficiencies typically range from η ≈ 0.65–0.80. Annual operating time may vary from intermittent operation (≈4000–6000 h/year) to continuous service (≈8760 h/year).

Within these realistic ranges, the absolute value of the annual energy saving may vary proportionally; however, the relative reduction in power demand associated with replacing steel flight bars by composite alternatives remains essentially unchanged, since it is governed primarily by the reduction in moving mass. Consequently, the order of magnitude of the energy saving—on the order of several thousand reais per year—remains robust across a wide range of industrial operating conditions.

For completeness and to facilitate international comparison, the annual energy costs were also converted to U.S. dollars using the 2019 average exchange rate of R$ 3.95 = US$ 1.00, yielding approximate annual costs of US$ 8185 for the steel configuration and US$ 5955 for the composite configurations.

The electricity tariff and exchange rate adopted in this study correspond to 2019 reference values and are intentionally used as a normalized baseline for comparative analysis. The objective of the energy assessment is not to predict absolute operational costs for a specific year, but rather to quantify the relative reduction in energy consumption resulting from flight-bar mass reduction. Since the annual energy cost scales linearly with electricity price and currency exchange rate, variations in these parameters do not affect the relative comparison between steel and composite configurations, nor the order of magnitude of the estimated savings.

Overall, the energy analysis demonstrates that replacing steel flight bars with polymer-matrix composite alternatives leads to a significant reduction in steady-state power demand and operational energy consumption. From a system-level perspective, this reduction creates the technical possibility of operating within a lower nominal power range or with reduced motor loading, while the final motor selection remains subject to conventional industrial design criteria and standardized equipment availability.

## 3. Results

### 3.1. Tensile Validation of the Glass/Epoxy Laminate

**Figure 6:**
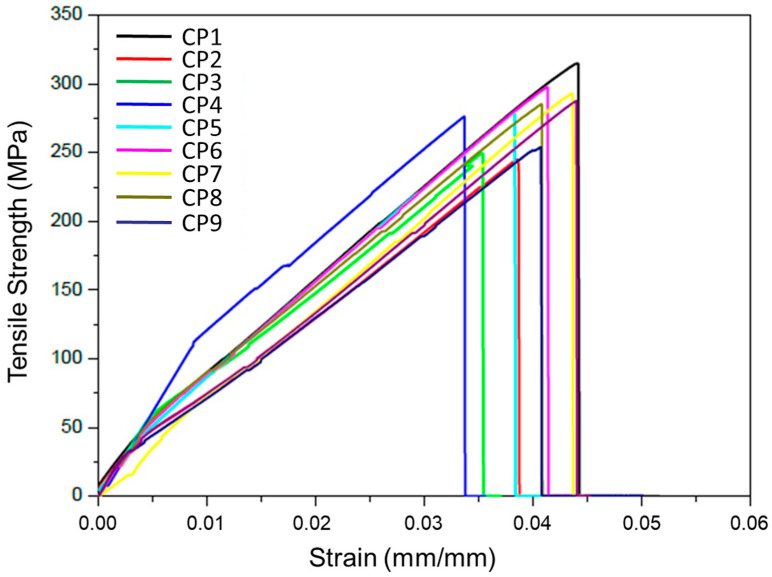
Stress–strain curves obtained from the tensile test of nine (9) specimens. One specimen exhibits a shorter curve due to premature failure, and no data smoothing, interpolation, or artificial correction was applied.

The tensile behavior of the vacuum-infused glass/epoxy laminate described in Section 2.7 is summarized in Table 4. Eight ASTM D3039 [26] specimens were tested, resulting in a tensile modulus of 25.0 ± 2.5 GPa, an ultimate tensile strength of 280 ± 22.7 MPa, and a failure strain of 2.4 ± 0.3%. The coefficients of variation (8–12%) fall within the expected range for woven E-glass laminates manufactured via vacuum infusion, reflecting characteristic variations in fiber volume fraction, tow compaction and resin-rich regions inherent to the process. These results confirm that the laminate fabricated for this study exhibits mechanical behavior consistent with that reported for similar woven E-glass/epoxy systems in the literature. The representative stress–strain response obtained from the tensile tests is shown in Figure 6.

It is important to note that the experimental tensile strength of the woven laminate (≈280 MPa) is not used to establish design allowable, since NBR 14574 [25] defines admissible stresses exclusively from unidirectional lamina properties. Therefore, the experimental values presented here serve solely to validate the analytical model and confirm the consistency of the micromechanical estimates adopted in this study.

### 3.2. Mechanical Assessment of the Glass/Epoxy Laminate

The tensile properties obtained experimentally for the vacuum-infused glass/epoxy laminate were incorporated into the analytical framework in order to reconcile the difference between handbook-based elastic properties and the measured mechanical response. As reported in Section 3.1, the laminate exhibited a tensile modulus of 25.0 ± 2.5 GPa and an ultimate tensile strength of 280 ± 22.7 MPa, which are significantly lower than the longitudinal modulus of 38 GPa typically reported for UD-equivalent glass/epoxy laminae in composite handbooks. This difference reflects fundamental microstructural and processing-related factors rather than inconsistencies in the analytical formulation.

To clarify this discrepancy, the fiber volume fraction of the experimental laminate was estimated from the measured constituent masses reported in Section 2.7. Considering a glass fiber mass of 257 g, an epoxy resin mass of 314.11 g, and typical densities of 2.55 g/cm^3^ for E-glass fibers and 1.15 g/cm^3^ for epoxy resin, the resulting fiber volume fraction is approximately V_f_ ≈ 0.27. This value is substantially lower than the fiber volume fractions commonly associated with UD-equivalent lamina properties reported in handbooks, which typically range from 0.45 to 0.60. When this experimentally inferred fiber volume fraction is introduced into classical micromechanical relations, such as the rule of mixtures, the expected longitudinal modulus falls within the range of 22–26 GPa, in excellent agreement with the experimentally measured tensile modulus. Additional reductions in effective stiffness arise from the bidirectional woven architecture and the vacuum infusion process, which introduce fiber crimp, tow undulation, resin-rich regions, and small void fractions.

It is important to emphasize that the elastic constants listed in Table 1 correspond to unidirectional-equivalent lamina properties used as input for Classical Laminate Theory (CLT), whereas the experimentally measured modulus represents the global tensile response of a woven laminate plate. These quantities are not expected to be numerically identical, as the former describes an idealized orthotropic lamina employed for analytical modeling, while the latter captures laminate-level averaging effects associated with fabric architecture and processing-induced heterogeneity.

**Figure 7:**
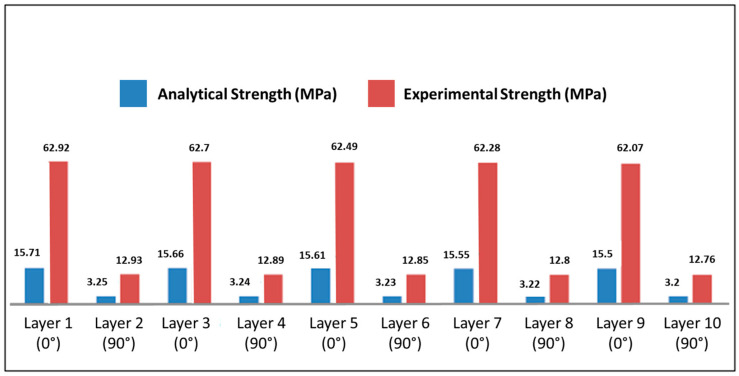
Graph of stress values in “X” per layer comparing stress values before and after mechanical characterization via tensile test.

To evaluate the influence of stiffness calibration on the structural predictions, a sensitivity update of the glass/epoxy lamina elastic properties was performed using the experimentally measured tensile modulus as reference. This update primarily affects the bending stiffness matrix [D] and, consequently, the predicted laminate curvature and deflection. However, the maximum ply-level stresses were found to be only weakly affected by this stiffness adjustment. This behavior does not arise from any ad hoc cancellation, but follows directly from the structure of Classical Laminate Theory under imposed bending moments. This effect is clearly illustrated in Figure 7, which compares the longitudinal stress distribution before and after stiffness calibration based on experimental tensile data.

In CLT, the laminate curvature vector is given by Equation (17):

(17) $$k={\left[D\right]}^{-1}M$$

while the ply-level stresses at a distance z_k_ from the laminate mid-plane are obtained as Equation (18):

(18) $$\sigma^{\left(k\right)}={\left[Q\right]}^{\left(k\right)}z_{k}\;k={\left[Q\right]}^{\left(k\right)}z_{k}\;{\left[D\right]}^{-1}M$$

Since the reduced stiffness matrix [Q]^(k)^ scales linearly with the elastic moduli of the lamina, whereas the bending stiffness matrix [D] scales with both the elastic moduli and the cube of the laminate thickness, the modulus dependence appears simultaneously in the numerator and denominator of the stress expression. As a result, for a given applied bending moment and fixed geometry, the stress magnitude is governed primarily by equilibrium and section lever-arm effects, while variations in elastic stiffness mainly affect curvature and deflection. This behavior is not specific to isotropic materials and remains valid for orthotropic laminates under linear elastic bending with zero bending–extension coupling.

Using the experimentally calibrated stiffness values, the predicted maximum longitudinal stress in the glass/epoxy flight bar remains on the order of approximately 95 MPa, with variations limited to only a few percent relative to the prediction based on handbook elastic constants. This stress level is therefore robust with respect to reasonable variations in elastic modulus and reflects the dominant role of applied bending moment and section geometry in determining ply-level stresses.

Finally, it is reiterated that the experimentally obtained tensile strength of the woven laminate is not used to redefine admissible design stresses, in accordance with the interpretation of ABNT NBR 14574 [25] discussed in Section 2.6. The experimental results serve to validate the micromechanical assumptions, reconcile stiffness discrepancies, and confirm the internal consistency of the multi-scale modeling framework adopted in this study. With these clarifications, the mechanical assessment of the glass/epoxy laminate is both physically sound and methodologically transparent.

### 3.3. Failure Index Evaluation—Maximum Stress and Azzi–Tsai–Hill Criteria

The structural integrity of the composite flight bars was evaluated at the ply level using stress-based failure assessments derived from the Classical Laminate Theory (CLT) results. The Maximum Stress criterion was applied to all laminate configurations—glass/epoxy, carbon/epoxy, and Kevlar/epoxy—while the Azzi–Tsai–Hill (ATH) interaction criterion was explicitly evaluated for the glass/epoxy system, for which experimental laminate strength data were available.

**Figure 8:**
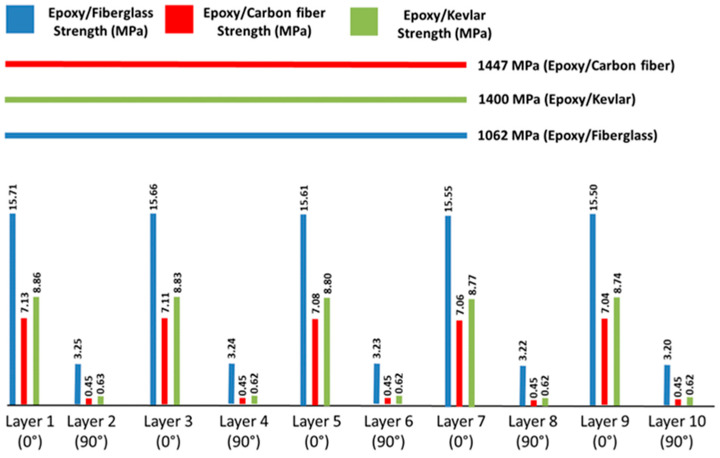
Graph of stress values in “X” per layer compared with maximum stress criterion.

For each ply of the equivalent laminate, the longitudinal (σ_1_), transverse (σ_2_), and in-plane shear (τ_12_) stresses were compared with the corresponding lamina-level strength parameters reported in Section 2.5.1. Under the operational bending moment of 342.02 N·m, none of the evaluated stress components exceeded their respective tensile, compressive, or shear strength limits for any of the composite systems investigated. According to the Maximum Stress criterion, no ply exhibited fiber-dominated, matrix-dominated, or shear-driven failure tendencies. Figure 8 compares the longitudinal stresses developed in the laminates with the corresponding Maximum Stress failure limits, confirming the absence of failure for all composite systems.

**Figure 9:**
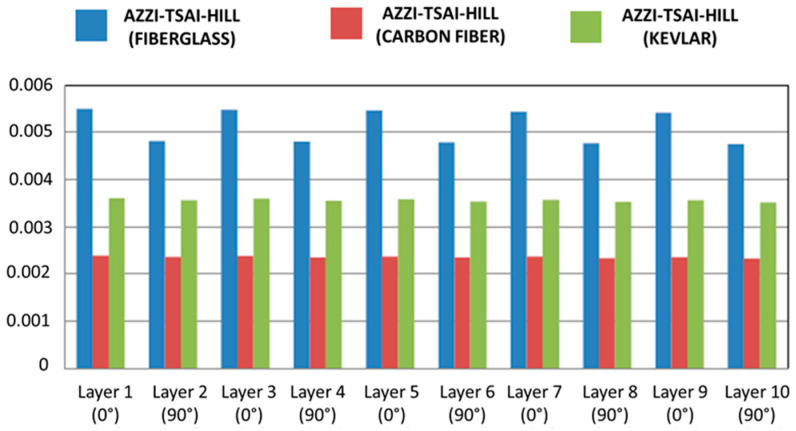
Graph of stress values in “X” per layer compared with the Azzi-Tsai-Hill criterion.

The Azzi–Tsai–Hill failure index distribution for the glass/epoxy laminate is shown in Figure 9.

For the glass/epoxy laminate, the Azzi–Tsai–Hill (ATH) criterion was additionally employed to account for the interaction between longitudinal, transverse, and shear stresses within the plies. The ATH failure index remained below unity for all plies, both when handbook-based elastic constants and experimentally calibrated stiffness values were used. Minor variations in the ATH index were observed due to stiffness calibration; however, these variations did not affect the overall safety assessment, and no ply approached the failure threshold.

For the carbon/epoxy and Kevlar/epoxy laminates, failure evaluation was restricted to the Maximum Stress criterion combined with conservative strength references obtained from composite materials handbooks. This approach avoids extrapolating interaction-based criteria beyond the material systems explicitly validated in this study and ensures methodological consistency in the comparative assessment.

In addition to the lamina-level failure criteria, a stress-reference verification was performed to contextualize the predicted service stresses with respect to established normative guidance. The Brazilian standard ABNT NBR 14574 [25] recommends, for preliminary structural design of polymer-matrix composite members, a conservative working-stress reference equal to 30% of the longitudinal tensile strength of a unidirectional (UD) lamina. For the glass/epoxy system, the UD lamina strength reported in the literature is Xt = 1062 MPa, yielding a UD-based benchmark of 0.30Xt = 318.6 MPa.

In the present study, this value is employed exclusively as a conservative UD-based benchmark for comparative purposes, and not as an admissible stress limit for woven laminates. This distinction is essential, as the flight bars analyzed herein are based on bidirectional woven fabrics (0°/90°), whose tensile strength is inherently lower than that of UD laminates due to fiber crimp, tow undulation, and resin-rich regions.

Accordingly, the structural feasibility of the glass/epoxy flight bar was verified by directly comparing the maximum longitudinal stress predicted under service loading with the experimentally measured tensile strength of the woven laminate. Tensile testing according to ASTM D3039 [26] yielded an experimental ultimate tensile strength of σᵤ,_exp_ ≈ 280 MPa (Section 3.1). The CLT results indicate a maximum longitudinal stress of approximately σ_x_,_max_ ≈ 95 MPa in the most highly solicited ply. This value is substantially lower than the experimental laminate strength, resulting in a margin of safety of approximately 2.95.

This comparison confirms that the glass/epoxy flight bar operates well within the elastic regime and below the experimentally observed failure limit of the woven laminate. It is also noted that the UD-based benchmark derived from ABNT NBR 14574 [25] exceeds the experimental laminate strength and therefore cannot be interpreted as an admissible stress for the woven system; its role in this study is limited to providing a conservative reference for contextualizing stress levels across different material systems.

For the carbon/epoxy and Kevlar/epoxy systems, experimental laminate strength data were not generated within the scope of this work. Therefore, strength references were taken from established composite materials handbooks and datasheets and were used consistently with the Maximum Stress criterion to provide a first-order feasibility assessment. In all cases, the predicted service stresses remained well below the selected strength references, indicating substantial safety margins under static bending.

Overall, the combined results of the stress-based failure criteria and the strength-reference verification demonstrate that all composite flight-bar configurations investigated are structurally feasible under the operational bending moment considered. Carbon/epoxy exhibits the lowest stress levels and the largest structural reserve, Kevlar/epoxy shows intermediate behavior consistent with its stiffness and density characteristics, and glass/epoxy—although more highly solicited—remains safely below its experimentally measured laminate strength.

The failure assessment presented in this study is intentionally restricted to static bending conditions and is based on stress-based initiation criteria, namely the Maximum Stress and Azzi–Tsai–Hill formulations. These criteria are widely employed in preliminary composite structural design to evaluate elastic feasibility and to identify critical stress states prior to more advanced damage analyses.

Fatigue behavior, progressive damage accumulation, interlaminar shear failure, and delamination mechanisms were not explicitly modeled. Although conveyor systems operate under repetitive loading cycles, the objective of the present work is not to predict service life or durability, but rather to establish whether the proposed composite flight bars operate well within the elastic regime under representative service bending moments.

The predicted ply-level stresses are significantly lower than the corresponding static strength limits for all investigated materials, which represents a necessary—though not sufficient—condition for satisfactory fatigue performance. A comprehensive reliability assessment would require fatigue testing, damage evolution models, and possibly cohesive-zone or progressive failure formulations, which are beyond the scope of this first-order feasibility study and are identified as essential topics for future work.

### 3.4. Motor Power Requirements and Energy Consumption

The substantial reduction in moving mass achieved by replacing steel flight bars with composite alternatives has a direct impact on the mechanical power required to operate the conveyor system under steady-state conditions. In the reference configuration, the 422 steel flight bars account for a total mass of 4.64 t, whereas the glass/epoxy, carbon/epoxy, and Kevlar/epoxy configurations reduce this value to 0.82 t, 0.74 t, and 0.68 t, respectively. This corresponds to a mass reduction of approximately 82–85% for the flight bars, which significantly lowers the total moving mass of the conveyor.

**Figure 10:**
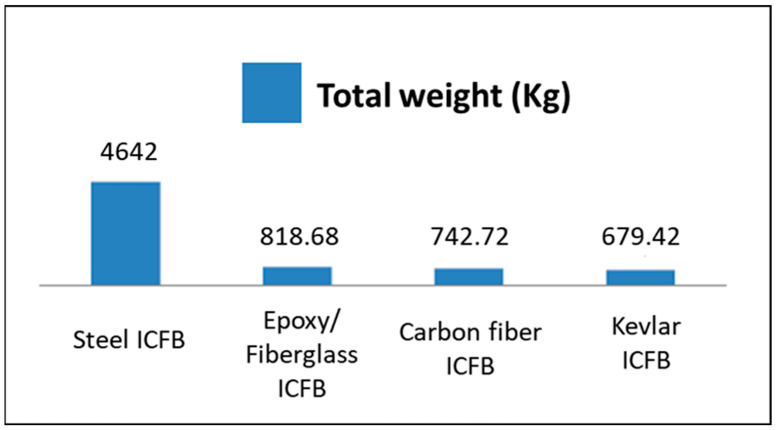
Graph of the masses corresponding to 422 slats made of steel, epoxy with fiberglass, epoxy with carbon fiber, and epoxy with Kevlar fiber.

Although the flight bars represent 48.7% of the total mass of the chain components, they do not account for the same fraction of the total moving mass of the conveyor, which also includes the transported load and other mechanical elements. The power values reported in this section were obtained by recalculating the total moving mass for each material configuration and subsequently recomputing the traction force and power demand using the full governing equations presented in Section 2.8. Figure 10 compares the total mass of the flight bars for the steel and composite configurations.

**Figure 11:**
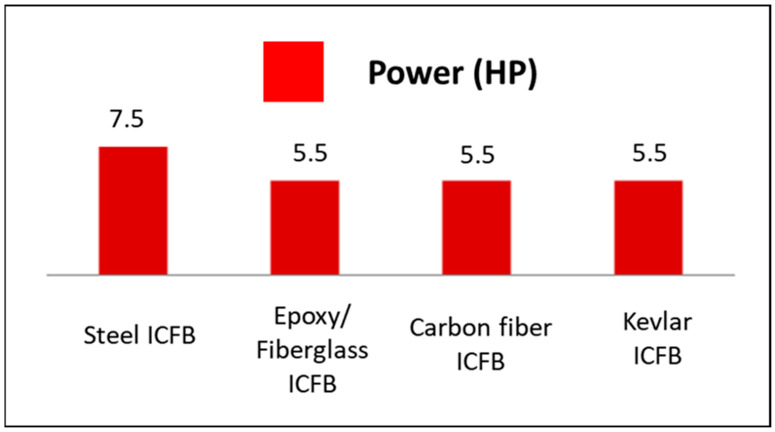
Graph of the power required to operate the conveyor with the 422 cleats made of steel, epoxy with fiberglass, epoxy with carbon fiber, and epoxy with Kevlar fiber.

As detailed in Section 2.8, the baseline steel-based system exhibits a calculated steady-state power demand of approximately 5.5 kW, corresponding to a nominal motor class of 7.5 CV. When composite flight bars are adopted, the reduced moving mass leads to a proportional decrease in the calculated mechanical power demand. For all three composite configurations, the resulting steady-state power requirement falls within a lower nominal power range, on the order of 4.0 kW, corresponding to a 5.5 CV motor class from an energetic standpoint. The resulting reduction in steady-state power requirements is illustrated in Figure 11.

It is important to emphasize that this reduction in calculated power demand does not imply that motor downsizing is automatic or mandatory in an industrial context. In practical conveyor systems, motor selection is influenced not only by steady-state power requirements, but also by starting torque, transient operating conditions, inertial effects, safety factors, gearbox characteristics, standardized motor ratings, and operational margins. Consequently, the reduction in power demand demonstrated here should be interpreted as placing the system within a lower nominal power range, thereby enabling either operation with reduced motor loading or the technical feasibility of selecting a lower-rated motor, subject to conventional industrial design criteria.

**Figure 12:**
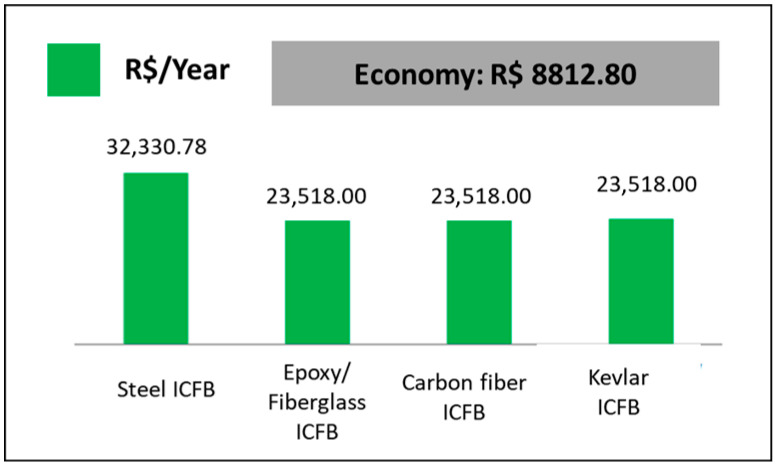
Graph of the financial expenditure on the electrical energy consumption of the conveyor according to the power required to operate the conveyor with the 422 cleats made of steel, epoxy with fiberglass, epoxy with carbon fiber, and epoxy with Kevlar fiber.

Assuming continuous operation of the conveyor system for 8760 h per year and adopting an industrial electricity tariff of R$ 0.671 per kWh, the annual energy consumption associated with the steel-based configuration is R$ 32,330.78. For the composite configurations, the corresponding annual energy cost is R$ 23,518.00 per year. The adoption of composite flight bars therefore results in an annual energy saving of R$ 8812.80, regardless of the specific composite reinforcement employed. Figure 12 summarizes the annual energy consumption cost for the steel and composite flight-bar configurations.

Table 5 summarizes the total mass of the flight bars, the nominal power range associated with steady-state operation, and the estimated annual energy costs for the steel and composite configurations. Although carbon/epoxy and Kevlar/epoxy flight bars provide slightly greater mass reduction than glass/epoxy, all composite alternatives fall within the same operational power range of the conveyor system. As a result, from an energy-consumption perspective, the three composite systems are equivalent, and material selection should be guided primarily by mechanical performance, durability requirements, manufacturing considerations, and lifecycle cost factors rather than by differences in steady-state energy demand alone.

Overall, the results demonstrate that replacing steel flight bars with polymer-matrix composite alternatives yields a significant reduction in steady-state mechanical power demand and operational energy consumption. From a system-level engineering perspective, this reduction improves operational efficiency and creates flexibility in motor loading and selection, while the final choice of installed motor power remains governed by standard industrial practices and project-specific constraints.

### 3.5. Comparative Structural Performance Summary

**Figure 13:**
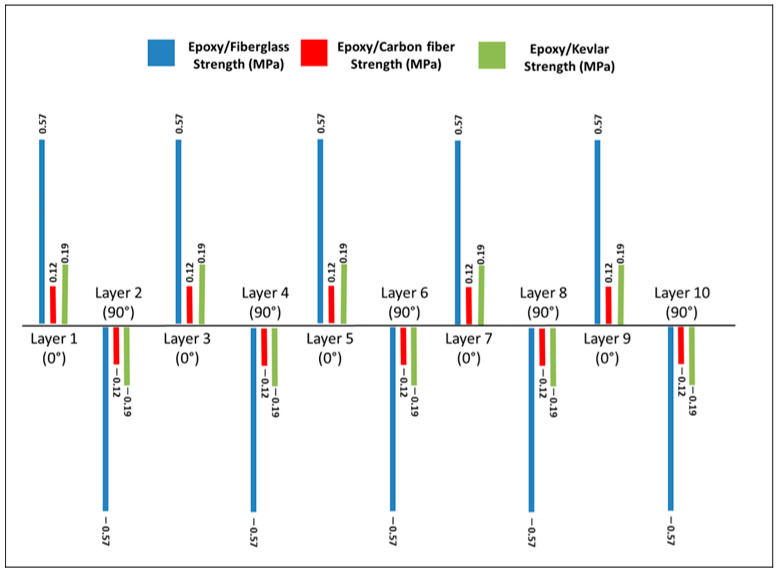
Graph of stress values in “Y” by layer and by composite type.

The structural analysis performed for the three composite systems—glass/epoxy, carbon/epoxy, and Kevlar/epoxy—reveals clear differences in bending response, stiffness distribution, and safety margins under the design bending moment of 342.02 N·m. Despite these differences, all laminate configurations were shown to be structurally feasible within the scope of the applied analytical framework. The corresponding transverse stress distributions across the laminate thickness are shown in Figure 13.

For all composite systems, ply-level stresses predicted by Classical Laminate Theory (CLT) satisfied the Maximum Stress failure criterion, with longitudinal, transverse, and in-plane shear stresses remaining below their respective lamina-level strength limits. In addition, the predicted service stresses were consistently lower than the conservative UD-based benchmark derived from ABNT NBR 14,574 [25], which was employed in this study exclusively for preliminary comparison purposes and not as an admissible design criterion for woven laminates. This dual verification provides a transparent reference context while preserving conceptual consistency with the laminate architecture investigated.

Carbon/epoxy laminates exhibited the lowest longitudinal stress levels among the evaluated materials, followed by Kevlar/epoxy and glass/epoxy laminates. This trend directly reflects the hierarchy of effective lamina stiffness and bending rigidity associated with the reinforcing fibers. The higher stiffness of carbon/epoxy results in reduced curvature under bending and consequently lower ply-level stresses, whereas glass/epoxy laminates, characterized by lower effective stiffness, experience higher stress levels under the same bending moment. Kevlar/epoxy laminates present intermediate behavior, consistent with their mechanical efficiency and lower density.

To enable a quantitative comparison of structural efficiency, two performance indices were defined based on the CLT results. The first index as Equation (19):

(19) $$\eta_{1}=\frac{\sigma_{x,max}}{\sigma_{tensile}}$$

represents the fraction of the laminate tensile capacity mobilized under service conditions, where σ_u_ is the reference tensile strength of the composite system. Lower values of η_1_ indicate larger structural safety margins. Based on this index, carbon/epoxy exhibits the lowest utilization ratio (*η*_1_ ≈ 0.06), reflecting a large mechanical reserve. Kevlar/epoxy presents an intermediate value (*η*_1_ ≈ 0.16), while glass/epoxy operates closer to its tensile capacity (*η*_1_ ≈ 0.34), though still within acceptable limits. The second index as Equation (20):

(20) $$\eta_{2}=\frac{\sigma_{x,max}}{\rho }$$

This provides a measure of specific solicitation normalized by density. Carbon/epoxy again presents the most favorable response (≈27 MPa·cm^3^/g), followed by Kevlar/epoxy (≈37 MPa·cm^3^/g) and glass/epoxy (≈49 MPa·cm^3^/g). These results indicate that carbon/epoxy offers the highest structural efficiency when both stress level and density are considered, whereas Kevlar/epoxy provides a balanced compromise between low density and moderate stress levels.

In addition to stress-based metrics, the mass reduction achieved with composite flight bars represents a critical performance indicator. Relative to the steel reference configuration (4642 kg for 422 bars), glass/epoxy, carbon/epoxy, and Kevlar/epoxy flight bars reduce the total mass to 819 kg, 743 kg, and 679 kg, respectively. Kevlar/epoxy therefore yields the highest mass reduction among the composite alternatives, followed by carbon/epoxy and glass/epoxy. These reductions directly influence system-level performance, as discussed in Section 4.5, by enabling a lower installed motor power without compromising structural safety.

Overall, the comparative results demonstrate that all three composite systems are capable of safely withstanding the operational bending moment imposed by the conveyor system. Carbon/epoxy provides the greatest structural reserve and the most favorable specific performance, Kevlar/epoxy achieves the largest mass reduction with intermediate stress levels, and glass/epoxy—although more heavily solicited—remains fully compliant with the applied design criteria. These findings establish a consistent and quantitative basis for material selection and for subsequent design optimization of composite flight bars in industrial conveyor applications. Table 6 presents a comparison of the structural and mass performance of the composite materials for the flight-bar.

## 4. Discussion

The structural and mechanical analyses carried out for the three composite systems—glass/epoxy, carbon/epoxy and Kevlar/epoxy—reveal important insights into the behavior of laminated composite flight bars subjected to bending loads representative of industrial conveyor operations. The layer wise stress results, failure-criterion evaluations and experimental validation collectively confirm the potential of composite materials to replace steel in this application, while also clarifying the distinct performance characteristics of each fiber reinforcement.

### 4.1. Influence of Fiber Type on Laminate Stiffness and Stress Distribution

The differences observed in the longitudinal and transverse stress distributions among the glass/epoxy, carbon/epoxy, and Kevlar/epoxy laminates are directly associated with the intrinsic stiffness hierarchy of their reinforcing fibers and with the way these properties are transferred to the laminate level through micromechanical homogenization. As highlighted by standard composite literature, carbon fibers at the fiber level exhibit elastic moduli typically exceeding 200–300 GPa, whereas E-glass fibers present moduli on the order of 70 GPa and aramid (Kevlar) fibers around 120–130 GPa. These values represent fundamental material properties of the reinforcements and should be distinguished from the effective elastic constants of composite laminae.

When these fibers are embedded in a polymer matrix and homogenized at the lamina level—accounting for fiber volume fraction, matrix properties, fiber–matrix interaction, and fabric architecture—this stiffness hierarchy is reflected in the effective longitudinal lamina moduli (E_L_) reported in Table 1. Accordingly, the carbon/epoxy system exhibits the highest lamina stiffness (E_L_ = 135 GPa), followed by Kevlar/epoxy (E_L_ = 71 GPa) and glass/epoxy (E_L_ = 38 GPa). The numerical differences between the fiber-level moduli cited above and the lamina-level elastic constants used in the Classical Laminate Theory (CLT) analysis are therefore physically consistent and expected, as the latter incorporate the compliance of the polymer matrix and the effects of composite architecture.

This hierarchy in lamina stiffness directly governs the bending response of the laminates. In the cross-ply stacking sequence adopted in this study, the bending stiffness is dominated by the contribution of the 0° plies, whose longitudinal elastic modulus plays a central role in determining the laminate curvature. As a result, the carbon/epoxy laminate exhibits the smallest mid-plane curvature under the applied bending moment, leading to the lowest ply-level longitudinal stresses. In contrast, the glass/epoxy laminate, characterized by the lowest effective lamina stiffness, undergoes larger curvature and consequently higher stress levels. Kevlar/epoxy laminates display intermediate behavior, consistent with their lamina-level modulus.

**Figure 14:**
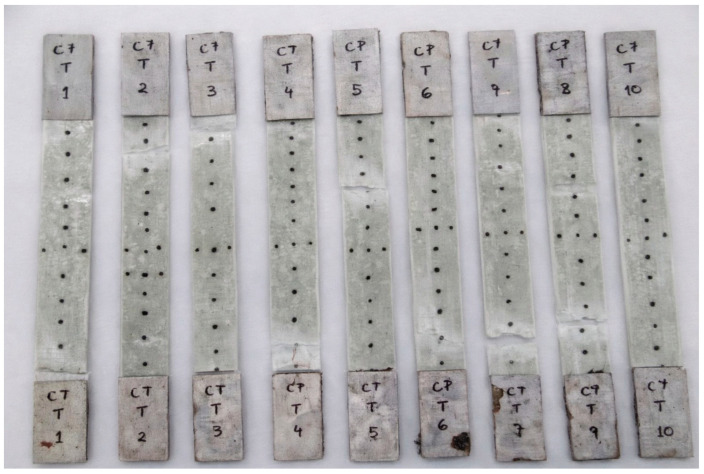
Failure mode observed in the glass/epoxy laminate after tensile testing, showing fracture in the gauge section.

These trends are fully consistent with classical laminate theory and with previous analytical and experimental studies on composite beams and plates subjected to bending loads. The results demonstrate that improvements in laminate stiffness and stress reduction are not solely a function of fiber stiffness at the material level, but rather of how these properties are transferred across scales—from fiber to lamina and from lamina to laminate—through micromechanical interactions and stacking-sequence design. In this context, the superior bending performance of carbon/epoxy laminates observed in the present study can be directly traced to the high stiffness of carbon fibers at the reinforcement scale and to their effective contribution to the longitudinal stiffness of the composite laminae. The failure mode observed in the glass/epoxy laminate after tensile testing is illustrated in Figure 14.

### 4.2. Influence of Fiber Morphology and Potential Failure Modes in Real Operation

The mechanical response observed for the three composite systems can also be interpreted in light of the intrinsic morphological characteristics of their reinforcing fibers. Carbon fibers exhibit a highly uniform and densely packed microstructure, with minimal surface defects, high graphitic alignment, and a low microfibril angle. These features contribute to their superior longitudinal stiffness (high E_L_), reduced susceptibility to shear deformation, and high compressive strength—properties that directly explain the lower longitudinal stresses and smaller failure indices observed in Section 3. In contrast, E-glass fibers present amorphous silica-based structures with higher inherent flaw populations, contributing to their lower modulus and strength, which is reflected in the higher ply stresses obtained for the glass/epoxy laminate. Kevlar fibers, characterized by their fibrillar, anisotropic morphology and strong hydrogen-bonded crystalline domains, provide high tensile performance but reduced compressive resistance due to micro buckling of fibrils—explaining the intermediate stiffness but more compliant bending response noted in the laminate-level results.

Although the present study focused primarily on elastic stress predictions and failure indices based on Classical Laminate Theory, it is important to recognize the potential failure mechanisms that may occur in real service conditions, particularly in components subjected to repeated bending and localized compressive loads such as flight bars in conveyor systems. Among these mechanisms, delamination is one of the most common, especially at the interfaces between 0° and 90° plies, where interlaminar shear stresses may accumulate under cyclic loading. Additionally, crushing due to compressive overloading, particularly in carbon and Kevlar systems, may occur when local compressive stresses exceed the micro buckling threshold of fibers. Interlaminar failure, including matrix cracking or interface debonding, may also develop in regions where transverse stresses (σ_y_) and shear stresses (τ_12_) interact, even if they remain below the allowable strength values predicted by the failure criteria [15,27].

While none of these failure modes were observed or experimentally induced in this work, acknowledging their relevance provides a more comprehensive interpretation of the laminate behavior. It also emphasizes the importance of combining numerical predictions with experimental evaluations—such as flexural testing, fatigue tests, or interlaminar fracture assessments (Mode I/II)—in future studies to fully characterize the durability and long-term performance of composite flight-bar structures. This consideration is particularly critical for Kevlar-based laminates, where the morphology associated with fibrillar splitting and low compressive stiffness may make the material more prone to out-of-plane deformation or shear-driven delamination under operational loads.

### 4.3. Transverse Response and Anisotropy Effects

For all three materials, transverse stresses (σγ) were found to be much lower than longitudinal stresses, typically representing less than 5% of the allowable strength—even for Kevlar, which has the lowest transverse mechanical performance. This is consistent with the classical behavior of cross-ply laminates under bending: transverse stresses are influenced primarily by Poisson effects and interply constraint rather than by direct bending loads.

The insignificance of σγ in the failure assessment indicates that the laminate design is not governed by transverse failure modes, a result also observed by [28] in composite naval components and by [29,30] in flexural studies of polymeric laminates. This confirms that the critical design direction for flight bars is the fiber-dominated (σ_x_) axis, which is adequately reinforced by the chosen stacking sequence.

### 4.4. Evaluation of Failure Criteria: Safety and Robustness

The structural safety of the composite flight bars was assessed using complementary failure criteria applied at different levels of rigor. For all laminate configurations—glass/epoxy, carbon/epoxy, and Kevlar/epoxy—the Maximum Stress criterion was employed to verify that the ply-level stress components remained below their respective allowable strength limits. In addition, the admissible working-stress threshold defined by ABNT NBR 14574 [25] was used as a conservative design benchmark for all materials.

Similar analytical approaches and design considerations for FRP systems in civil engineering—covering durability aspects and alternative reinforcement concepts—have been discussed in the literature [31,32].

An additional level of verification was performed for the glass/epoxy laminate through the explicit application of the Azzi–Tsai–Hill (ATH) quadratic failure criterion. This criterion accounts for the interaction between longitudinal, transverse, and shear stresses and therefore provides a more comprehensive assessment of multidimensional stress states. The ATH failure indices obtained for the glass/epoxy laminate remained below unity for all plies, further confirming the robustness of the structural assessment.

The combined use of these criteria ensures a conservative and transparent evaluation of laminate safety while maintaining methodological consistency with the scope of the analyses explicitly demonstrated in the Results section.

### 4.5. Practical Implications: Weight Reduction and Energy Efficiency

One of the most significant practical outcomes of this study concerns the reduction in energy consumption enabled by replacing conventional steel flight bars with polymer-matrix composite alternatives. In the baseline configuration, the 422 steel flight bars account for a total mass of 4.64 t and require an installed motor power of 7.5 CV (5.5 kW) to operate the conveyor system under continuous service conditions. When composite flight bars are adopted, the substantial reduction in moving mass—ranging from approximately 82% to 85%—allows the system to operate safely with an installed power of 5.5 CV (4.0 kW).

Among the composite alternatives, Kevlar/epoxy provides the lowest total flight-bar mass (679 kg), followed by carbon/epoxy (743 kg) and glass/epoxy (819 kg). Although these materials differ in density, stiffness, and mass reduction, all composite configurations fall below the same operational power threshold of the conveyor system. Consequently, the reduction in installed motor power is identical for the three composite solutions and is governed by system-level constraints rather than by marginal differences in composite density.

Assuming continuous operation of 8760 h per year and an industrial electricity tariff of R$ 0.671 per kWh, the annual energy cost associated with the steel-based configuration is R$ 32,330.78. In contrast, all composite configurations result in an annual energy cost of R$ 23,518.00, yielding an annual saving of approximately R$ 8812.80. This reduction in operational energy expenditure represents a direct and quantifiable benefit of mass substitution at the system level and is independent of the specific composite reinforcement employed.

It is important to emphasize that the economic analysis presented in this work is restricted to operational energy consumption (OPEX). No quantitative assessment of material costs, manufacturing processes, or initial investment (CAPEX) was performed. Therefore, while the results clearly demonstrate an energetic advantage associated with the use of composite flight bars, a comprehensive cost comparison between steel and composite solutions—including material price, fabrication costs, and lifecycle economics—remains outside the scope of the present study and should be addressed in future investigations.

From an engineering design perspective, these findings indicate that the selection of composite reinforcement for industrial conveyor flight bars should be guided primarily by mechanical performance, durability requirements, damage tolerance, and manufacturing considerations, rather than by energy efficiency alone. Carbon/epoxy laminates offer the highest stiffness and largest structural safety margins, Kevlar/epoxy laminates provide the greatest mass reduction and may be advantageous where toughness is critical, and glass/epoxy laminates remain a structurally viable alternative with moderate mass reduction. Together, these results demonstrate that polymer-matrix composites can deliver substantial operational energy savings while maintaining structural integrity in heavy-duty conveyor systems, even when economic considerations beyond energy consumption are not explicitly evaluated.

### 4.6. Design Considerations for Industrial Implementation

The results obtained in this study highlight several important considerations for the practical implementation of composite flight bars in industrial conveyor systems. From a structural standpoint, all composite configurations investigated—glass/epoxy, carbon/epoxy, and Kevlar/epoxy—are capable of safely withstanding the operational bending moment imposed by the conveyor, as verified through ply-level stress analysis, the Maximum Stress criterion, and the admissible limits prescribed by ABNT NBR 14574 [25]. These findings confirm that polymer-matrix composites can be considered technically viable substitutes for steel flight bars in load-bearing conveyor applications.

Material selection for industrial implementation should primarily be guided by mechanical performance requirements. Carbon/epoxy laminates exhibited the highest longitudinal stiffness and the largest structural safety margins, making them particularly suitable for applications where minimal deformation and high bending rigidity are critical. Kevlar/epoxy laminates, while less stiff, provided the lowest overall mass and may offer advantages in situations where reduced inertia, enhanced toughness, or impact resistance are relevant. Glass/epoxy laminates, although presenting higher stress levels than the other composite systems, remained fully compliant with all applied design criteria and represent a structurally viable option where moderate mass reduction and material availability are prioritized.

From an operational perspective, all composite configurations produced the same reduction in installed motor power and identical annual energy savings, as discussed in Section 4.5. Consequently, differences in energy efficiency do not constitute a discriminating factor among the composite systems investigated. Instead, once the system-level power reduction threshold is reached, the choice of reinforcement should be based on structural efficiency, durability, environmental resistance, and manufacturing constraints rather than on operational energy considerations alone.

Manufacturability is another critical aspect for industrial adoption. The laminate architectures considered in this study are compatible with established composite manufacturing techniques such as vacuum infusion, which enables the production of relatively large and geometrically simple components with consistent quality. However, the industrial deployment of composite flight bars would require additional evaluation of process repeatability, quality control, and scalability, particularly for high-volume production environments. Factors such as fiber handling, curing cycles, dimensional tolerances, and integration with metallic interfaces (e.g., pins, bushings, and fastening regions) should be carefully addressed during detailed design stages.

Finally, it is important to note that the present work did not include a quantitative assessment of material costs, fabrication expenses, or initial investment (CAPEX). As a result, no ranking of composite systems in terms of overall economic performance can be established based on the current dataset. Future studies should therefore incorporate comprehensive cost analyses, including material pricing, manufacturing costs, lifecycle considerations, and potential maintenance savings, in order to support informed decision-making regarding large-scale industrial implementation of composite flight bars.

### 4.7. Limitations and Future Work

The present study provides a first-order structural and energetic feasibility assessment of composite flight bars for industrial conveyor systems. While the adopted methodology integrates micromechanical modeling, Classical Laminate Theory (CLT), stress-based failure criteria, and targeted experimental validation, several limitations must be acknowledged to properly contextualize the scope and applicability of the results.

First, the experimental validation was intentionally restricted to tensile testing of a vacuum-infused glass/epoxy laminate. This material was selected as a validation anchor because it represents the mechanically most critical configuration among the investigated composites, exhibiting the lowest stiffness and the highest predicted stress levels under the same bending moment. Carbon/epoxy and Kevlar/epoxy systems were evaluated exclusively using micromechanical estimates and well-established handbook properties. While this approach is consistent with standard preliminary composite structural design practice, it does not capture manufacturing-specific effects, fiber architecture variability, or material-dependent defects that may arise in real components.

Second, the composite flight bar was modeled as an equivalent fully laminated solid section occupying the entire 60 mm × 20 mm cross section. This representation was adopted to preserve geometric compatibility with the steel reference component and to ensure consistency between structural analysis, mass estimation, and energy-consumption evaluation. In practical manufacturing, a composite component of this thickness would not be produced as a single monolithic laminate in one processing step, but rather through multi-stage vacuum infusion, sub-laminate co-curing, prepreg layup with intermediate debulking, resin transfer molding, pultrusion, or similar industrial routes. Manufacturing-induced phenomena such as void content, resin-rich regions, thickness-dependent consolidation effects, and potential delamination were therefore not explicitly modeled and should be addressed through dedicated experimental studies.

Third, the structural analysis was based on Classical Laminate Theory and restricted to linear elastic behavior under static bending loads. Higher-order effects, including transverse shear deformation, interlaminar stress concentrations, and local stress gradients near supports or load introduction regions, were not explicitly considered. Although the high slenderness ratio of the flight bar (L/h ≈ 48) justifies the use of CLT for global stress prediction, more refined analyses employing higher-order laminate theories or three-dimensional finite element modeling would be required to capture localized phenomena and boundary effects with greater fidelity.

Fourth, the failure assessment relied exclusively on stress-based initiation criteria, namely the Maximum Stress and Azzi–Tsai–Hill formulations. These criteria are appropriate for evaluating elastic feasibility and identifying critical stress states but do not account for fatigue behavior, progressive damage accumulation, interlaminar shear failure, or delamination mechanisms. Since conveyor systems operate under repetitive loading cycles, a comprehensive durability and reliability assessment would require fatigue testing, damage evolution models, and possibly cohesive-zone or progressive failure approaches, which are beyond the scope of the present feasibility study.

Fifth, the strength properties employed in the failure criteria include unidirectional (UD) lamina strength values used as reference limits for woven bidirectional laminates. This approach, commonly adopted in preliminary CLT-based analyses, enables standardized comparison of ply-level stress states but may overestimate the actual strength of woven laminates if interpreted as admissible design values, due to fiber crimp, tow undulation, and resin-rich regions. This limitation was explicitly mitigated for the glass/epoxy system by directly comparing predicted service stresses with the experimentally measured tensile strength of the woven laminate. For the carbon/epoxy and Kevlar/epoxy systems, the results should be interpreted as first-order feasibility indicators rather than definitive design allowable.

Finally, the energy-consumption analysis was conducted using reference electricity tariffs and exchange rates adopted as a normalized baseline for comparative purposes. While absolute monetary values may vary with time, location, and operating conditions, the relative reduction in power demand and energy consumption demonstrated in this study is governed primarily by the reduction in moving mass and therefore remains robust across realistic industrial parameter ranges.

Future work should focus on experimental fabrication and testing of full-scale composite flight bars, including flexural, fatigue, and interlaminar characterization, as well as detailed finite element simulations to assess localized stress states, boundary effects, and damage initiation. In addition, manufacturing process optimization, cost analysis, and long-term durability assessment under representative service conditions are essential steps toward full industrial qualification and implementation of composite flight bars in heavy-duty conveyor systems.

## 5. Conclusions

Based on the structural and energetic assessment performed in this study, the following conclusions can be drawn:

1. A multiscale analytical framework combining micromechanical modeling, Classical Laminate Theory (CLT), ply-level failure criteria, and experimental tensile validation was successfully applied to evaluate composite flight bars for industrial conveyor systems under a representative bending moment.
2. All composite configurations investigated—glass/epoxy, carbon/epoxy, and Kevlar/epoxy—were shown to be structurally feasible under the applied bending load. Ply-level stresses remained well below the corresponding strength limits according to the Maximum Stress criterion, and, for the glass/epoxy system, also satisfied the Azzi–Tsai–Hill interaction criterion.
3. The Brazilian standard ABNT NBR 14574 was employed exclusively as a comparative benchmark based on unidirectional lamina properties and not as an admissible design limit for the bidirectional woven laminates analyzed. Structural feasibility was therefore established through stress-based failure criteria and, for the glass/epoxy laminate, by direct comparison with experimentally measured tensile strength.
4. Among the materials evaluated, carbon/epoxy laminates exhibited the lowest longitudinal stress levels and the largest structural reserve due to their superior bending stiffness. Kevlar/epoxy laminates presented intermediate behavior, combining low density with moderate stress levels, while glass/epoxy laminates—although more highly solicited—remained safely below their experimental failure limits.
5. Sensitivity analysis using experimentally calibrated elastic properties for the glass/epoxy system showed that variations in elastic modulus primarily affect laminate curvature and deflection, whereas ply-level stress predictions remain weakly affected. This behavior follows directly from the structure of CLT under imposed bending moments.
6. From a system-level perspective, replacing steel flight bars with composite alternatives resulted in an 82–85% reduction in flight-bar mass, leading to a significant decrease in steady-state mechanical power demand. The calculated power requirement falls within a lower nominal motor class, yielding an estimated annual energy saving of approximately R$ 8812.80 under continuous operation.
7. Although the reduced power demand does not imply automatic motor downsizing, the results demonstrate that composite flight bars reduce operational energy consumption and enable operation with reduced motor loading, subject to standard industrial design criteria.
8. Overall, the study demonstrates that polymer-matrix composite flight bars constitute a structurally safe and energetically advantageous alternative to steel components in industrial conveyor systems, with carbon/epoxy emerging as the most mechanically efficient solution among the materials investigated.

## Figures and Tables

**Table 1:** Lamina-level elastic properties adopted as input for the Classical Laminate Theory (CLT) analysis.

Material System	E_L_ (GPa)	E_T_ (GPa)	G_LT_ (GPa)	v_LT_	References
Glass/Epoxy	38	8.4	4.5	0.26	[18]
Glass/Epoxy	25	8.4	4.5	0.26	This work
Carbon/Epoxy	135	10	5.0	0.28	[19]
Kevlar/Epoxy	71	5	2.5	0.34	[19,20]

**Table 2:** Geometric and sectional parameters adopted for the composite flight-bar structural analysis.

Parameter	Symbol	Value	Note
Bar width	b	60 mm	Identical to the steel reference flight bar
Bar height	h	20 mm	Entire cross section assumed to be load-bearing composite
Cross-section type	-	Rectangular	Same external geometry as steel component
Laminate idealization	-	Fully laminated solid section	Equivalent laminate representation
Stacking sequence (conceptual)	-	[0/90]^N^	N sufficiently large to reproduce 20 mm thickness
Reference ply thickness	t_ply_	0.32 mm	Based on experimental VIP glass/epoxy laminate
Reference number of plies (calibration laminate)	N_ref_	10	Used only for micromechanical calibration
Reference laminate thickness (calibration)	t_ref_	3.20 mm	Not the structural thickness of the flight bar
Structural laminate thickness used in CLT	_tlam_	20 mm	Thickness used for *z* z-coordinate and [D] matrix
Distance to outer fibers	z_max_	±10 mm	Consistent with full section height
Applied bending moment	M	342.02 N·m	Same design load as steel reference
Bending moment per unit width	M_x_	5.70 N·m/mm	M_x_ = M/b
Steel section modulus (reference)	W_steel_	10,882 mm^3^	Used for baseline comparison
Steel moment of inertia (reference)	I_steel_	108,820 mm^4^	I = W × h/2
Stress evaluation method	-	Classical Laminate Theory (CLT)	Ply-wise stress integration
Mass calculation assumption	-	Full composite section (60 × 20 mm^2^)	No core or non-structural filler

**Table 3:** Lamina strength properties used in the Maximum Stress and Azzi–Tsai–Hill failure criteria.

Material System	Xt (MPa)	Xc (MPa)	Yt (MPa)	Yc (MPa)	S (MPa)	References
Glass/Epoxy	1062	610	31	118	72	[18]
Carbon/Epoxy	1500	1200	40	246	68	[19]
Kevlar/Epoxy	1400	250	20	97	41	[19,20]

**Table 4:** Tensile properties of the glass/epoxy laminate.

Property	Mean	Standard Deviation	COV (%)
Tensile modulus, E (GPa)	25.0	2.5	10%
Ultimate tensile strength, σ_u_ (MPa)	280	22.7	8.1%
Ultimate strain, ε_u_ (%)	2.4	0.3	12%

**Table 5:** Mass and energy comparison between materials.

System	Total Mass of 422 Bars (kg)	Required Motor Power	Annual Energy Cost (R$)	Annual Savings vs. Steel (R$)
Steel (baseline)	4642	7.5 CV (5.5 kW)	32,330.78	-
Glass/Epoxy	819	5.5 CV (4.0 kW)	23,518.00	8812.80
Carbon/Epoxy	743	5.5 CV (4.0 kW)	23,518.00	8812.80
Kevlar/Epoxy	679	5.5 CV (4.0 kW)	23,518.00	8812.80

Note: Annual energy costs were calculated assuming continuous operation (8760 h/year) and an industrial electricity tariff of R$ 0.671/kWh. Motor power values are reported both in CV and kW for clarity.

**Table 6:** Comparative structural and mass performance of the composite flight-bar materials.

Material	Density (g/cm^3^)	Laminate Tensile Strength (MPa)	σ_x_,_max_ (MPa)	η_1_ = σ_x_,_max_/Strength	η_2_ = σ_x_,_max_/Density (MPa·cm^3^/g)	Notes
Glass/Epoxy	1.95	280 (experimental)	95	0.34	48.7	Baseline laminate; highest solicitation
Carbon/Epoxy	1.60	600–800 [19]	43	0.06	26.9	Most efficient; largest structural reserve
Kevlar/Epoxy	1.44	300–350 [20]	53	0.16	36.8	Lowest density; intermediate efficiency

## Data Availability

The original contributions presented in the study are included in the article; further inquiries can be directed to the corresponding author.
